# Learning strategies: a synthesis and conceptual model

**DOI:** 10.1038/npjscilearn.2016.13

**Published:** 2016-08-10

**Authors:** John A C Hattie, Gregory M Donoghue

**Affiliations:** 1 Science of Learning Research Centre, Graduate School of Education, University of Melbourne, Carlton, VIC, Australia

## Abstract

The purpose of this article is to explore a model of learning that proposes that various learning strategies are powerful at certain stages in the learning cycle. The model describes three inputs and outcomes (skill, will and thrill), success criteria, three phases of learning (surface, deep and transfer) and an acquiring and consolidation phase within each of the surface and deep phases. A synthesis of 228 meta-analyses led to the identification of the most effective strategies. The results indicate that there is a subset of strategies that are effective, but this effectiveness depends on the phase of the model in which they are implemented. Further, it is best not to run separate sessions on learning strategies but to embed the various strategies within the content of the subject, to be clearer about developing both surface and deep learning, and promoting their associated optimal strategies and to teach the skills of transfer of learning. The article concludes with a discussion of questions raised by the model that need further research.

There has been a long debate about the purpose of schooling. These debates include claims that schooling is about passing on core notions of humanity and civilisation (or at least one’s own society’s view of these matters). They include claims that schooling should prepare students to live pragmatically and immediately in their current environment, should prepare students for the work force, should equip students to live independently, to participate in the life of their community, to learn to ‘give back’, to develop personal growth.^1^

In the past 30 years, however, the emphasis in many western systems of education has been more on enhancing academic achievement—in domains such as reading, mathematics, and science—as the primary purpose of schooling.^2^ Such an emphasis has led to curricula being increasingly based on achievement in a few privileged domains, and ‘great’ students are deemed those who attain high levels of proficiency in these narrow domains.

This has led to many countries aiming to be in the top echelon of worldwide achievement measures in a narrow range of subjects; for example, achievement measures such as PISA (tests of 15-year olds in mathematics, reading and science, across 65 countries in 2012) or PIRLS (Year-5 tests of mathematics, reading and science, across 57 countries in 2011). Indeed, within most school systems there is a plethora of achievement tests; many countries have introduced accountability pressures based on high levels of testing of achievement; and communities typically value high achievement or levels of knowledge.^3^ The mantra underpinning these claims has been cast in terms of what students know and are able to do; the curriculum is compartmentalised into various disciplines of achievement; and students, teachers, parents and policy makers talk in terms of success in these achievement domains.

Despite the recent emphasis on achievement, the day-to-day focus of schools has always been on learning—how to know, how to know more efficiently and how to know more effectively. The underlying philosophy is more about what students are now ready to learn, how their learning can be enabled, and increasing the ‘how to learn’ proficiencies of students. In this scenario, the purpose of schooling is to equip students with learning strategies, or the skills of learning how to learn. Of course, learning and achievement are not dichotomous; they are related.^4^ Through growth in learning in specific domains comes achievement and from achievement there can be much learning. The question in this article relates to identifying the most effective strategies for learning.

In our search, we identified >400 learning strategies: that is, those processes which learners use to enhance their own learning. Many were relabelled versions of others, some were minor modifications of others, but there remained many contenders purported to be powerful learning strategies. Such strategies help the learner structure his or her thinking so as to plan, set goals and monitor progress, make adjustments, and evaluate the process of learning and the outcomes. These strategies can be categorised in many ways according to various taxonomies and classifications (e.g., references 5,6,7). Boekaerts,^8^ for example, argued for three types of learning strategies: (1) cognitive strategies such as elaboration, to deepen the understanding of the domain studied; (2) metacognitive strategies such as planning, to regulate the learning process; and (3) motivational strategies such as self-efficacy, to motivate oneself to engage in learning. Given the advent of newer ways to access information (e.g., the internet) and the mountain of information now at students’ fingertips, it is appropriate that Dignath, Buettner and Langfeldt^9^ added a fourth category—management strategies such as finding, navigating, and evaluating resources.

But merely investigating these 400-plus strategies as if they were independent is not defensible. Thus, we begin with the development of a model of learning to provide a basis for interpreting the evidence from our meta-synthesis. The argument is that learning strategies can most effectively enhance performance when they are matched to the requirements of tasks (cf.^10^).

## A model of learning

**Figure 1:**
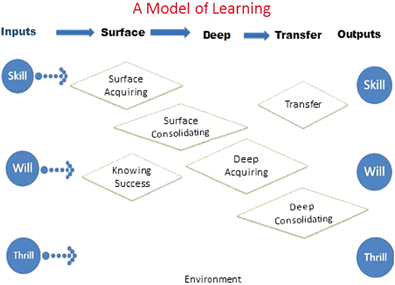
A model of learning.

The model comprises the following components: three inputs and three outcomes; student knowledge of the success criteria for the task; three phases of the learning process (surface, deep and transfer), with surface and deep learning each comprising an acquisition phase and a consolidation phase; and an environment for the learning (Figure 1). We are proposing that various learning strategies are differentially effective depending on the degree to which the students are aware of the criteria of success, on the phases of learning process in which the strategies are used, and on whether the student is acquiring or consolidating their understanding. The following provides an overview of the components of the model (see reference 11 for a more detailed explanation of the model).

### Input and outcomes

The model starts with three major sources of inputs: the skill, the will and the thrill. The ‘skill’ is the student’s prior or subsequent achievement, the ‘will’ relates to the student’s various dispositions towards learning, and the ‘thrill’ refers to the motivations held by the student. In our model, these inputs are also the major outcomes of learning. That is, developing outcomes in achievement (skill) is as valuable as enhancing the dispositions towards learning (will) and as valuable as inviting students to reinvest more into their mastery of learning (thrill or motivations).

#### The skill

The first component describes the prior achievement the student brings to the task. As Ausubel^12^ claimed ‘if I had to reduce all of educational psychology to just one principle, I would say this ‘The most important single factor influencing learning is what the leaner already knows. Ascertain this and teach him accordingly. Other influences related to the skills students bring to learning include their working memory, beliefs, encouragement and expectations from the student’s cultural background and home.

#### The will

Dispositions are more habits of mind or tendencies to respond to situations in certain ways. Claxton^13^ claimed that the mind frame of a ‘powerful learner’ is based on the four major dispositions: resilience or emotional strength, resourcefulness or cognitive capabilities, reflection or strategic awareness, and relating or social sophistication. These dispositions involve the proficiency to edit, select, adapt and respond to the environment in a recurrent, characteristic manner.^14^ But dispositions alone are not enough. Perkins *et al.* ^15^ outlined a model with three psychological components which must be present in order to spark dispositional behaviour: sensitivity—the perception of the appropriateness of a particular behaviour; inclination—the felt impetus toward a behaviour; and ability—the basic capacity and confidence to follow through with the behaviour.

#### The thrill

There can be a thrill in learning but for many students, learning in some domains can be dull, uninviting and boring. There is a huge literature on various motivational aspects of learning, and a smaller literature on how the more effective motivational aspects can be taught. A typical demarcation is between mastery and performance orientations. Mastery goals are seen as being associated with intellectual development, the acquisition of knowledge and new skills, investment of greater effort, and higher-order cognitive strategies and learning outcomes.^16^ Performance goals, on the other hand, have a focus on outperforming others or completing tasks to please others. A further distinction has been made between approach and avoidance performance goals.^17–19^ The correlations of mastery and performance goals with achievement, however, are not as high as many have claimed. A recent meta-analysis found 48 studies relating goals to achievement (based on 12,466 students), and the overall correlation was 0.12 for mastery and 0.05 for performance goals on outcomes.^20^ Similarly, Hulleman *et al.* ^21^ reviewed 249 studies (*N*=91,087) and found an overall correlation between mastery goal and outcomes of 0.05 and performance goals and outcomes of 0.14. These are small effects and show the relatively low importance of these motivational attributes in relation to academic achievement.

An alternative model of motivation is based on Biggs^22^ learning processes model, which combines motivation (why the student wants to study the task) and their related strategies (how the student approaches the task). He outlined three common approaches to learning: deep, surface and achieving. When students are taking a deep strategy, they aim to develop understanding and make sense of what they are learning, and create meaning and make ideas their own. This means they focus on the meaning of what they are learning, aim to develop their own understanding, relate ideas together and make connections with previous experiences, ask themselves questions about what they are learning, discuss their ideas with others and compare different perspectives. When students are taking a surface strategy, they aim to reproduce information and learn the facts and ideas—with little recourse to seeing relations or connections between ideas. When students are using an achieving strategy, they use a ‘minimax’ notion—minimum amount of effort for maximum return in terms of passing tests, complying with instructions, and operating strategically to meet a desired grade. It is the achieving strategy that seems most related to school outcomes.

### Success criteria

The model includes a prelearning phase relating to whether the students are aware of the criteria of success in the learning task. This phase is less about whether the student desires to attain the target of the learning (which is more about motivation), but whether he or she understands what it means to be successful at the task at hand. When a student is aware of what it means to be successful before undertaking the task, this awareness leads to more goal-directed behaviours. Students who can articulate or are taught these success criteria are more likely to be strategic in their choice of learning strategies, more likely to enjoy the thrill of success in learning, and more likely to reinvest in attaining even more success criteria.

Success criteria can be taught.^23,24^ Teachers can help students understand the criteria used for judging the students’ work, and thus teachers need to be clear about the criteria used to determine whether the learning intentions have been successfully achieved. Too often students may know the learning intention, but do not how the teacher is going to judge their performance, or how the teacher knows when or whether students have been successful.^25^ The success criteria need to be as clear and specific as possible (at surface, deep, or transfer level) as this enables the teacher (and learner) to monitor progress throughout the lesson to make sure students understand and, as far as possible, attain the intended notions of success. Learning strategies that help students get an overview of what success looks like include planning and prediction, having intentions to implement goals, setting standards for judgement success, advance organisers, high levels of commitment to achieve success, and knowing about worked examples of what success looks like.^23^

### Environment

Underlying all components in the model is the environment in which the student is studying. Many books and internet sites on study skills claim that it is important to attend to various features of the environment such as a quiet room, no music or television, high levels of social support, giving students control over their learning, allowing students to study at preferred times of the day and ensuring sufficient sleep and exercise.

### The three phases of learning: surface, deep and transfer

The model highlights the importance of both surface and deep learning and does not privilege one over the other, but rather insists that both are critical. Although the model does seem to imply an order, it must be noted that these are fuzzy distinctions (surface and deep learning can be accomplished simultaneously), but it is useful to separate them to identify the most effective learning strategies. More often than not, a student must have sufficient surface knowledge before moving to deep learning and then to the transfer of these understandings. As Entwistle^26^ noted, ‘The verb ‘to learn’ takes the accusative’ that is, it only makes sense to analyse learning in relation to the subject or content area and the particular piece of work towards which the learning is directed, and also the context within which the learning takes place. The key debate, therefore, is whether the learning is directed content that is meaningful to the student, as this will directly affect student dispositions, in particular a student’s motivation to learn and willingness to reinvest in their learning.

A most powerful model to illustrate this distinction between surface and deep is the structure of observed learning outcomes, or SOLO,^27,28^ as discussed above. The model has four levels: unistructural, multistructural, relational and extended abstract. A unistructural intervention is based on teaching or learning one idea, such as coaching one algorithm, training in underlining, using a mnemonic or anxiety reduction. The essential feature is that this idea alone is the focus, independent of the context or its adaption to or modification by content. A multistructural intervention involves a range of independent strategies or procedures, but without integrating or orchestration as to the individual differences or demands of content or context (such as teaching time management, note taking and setting goals with no attention to any strategic or higher-order understandings of these many techniques). Relational interventions involve bringing together these various multistructural ideas, and seeing patterns; it can involve the strategies of self-monitoring and self-regulation. Extended abstract interventions aim at far transfer (transfer between contexts that, initally, appear remote to one another) such that they produce structural changes in an individual’s cognitive functioning to the point where autonomous or independent learning can occur. The first two levels (one then many ideas) refer to developing surface knowing and the latter two levels (relate and extend) refer to developing deeper knowing. The parallel in learning strategies is that surface learning refers to studying without much reflecting on either purpose or strategy, learning many ideas without necessarily relating them and memorising facts and procedures routinely. Deep learning refers to seeking meaning, relating and extending ideas, looking for patterns and underlying principles, checking evidence and relating it to conclusions, examining arguments cautiously and critically, and becoming actively interested in course content (see reference 29).

Our model also makes a distinction between first acquiring knowledge and then consolidating it. During the acquisition phase, information from a teacher or instructional materials is attended to by the student and this is taken into short-term memory. During the consolidation phase, a learner then needs to actively process and rehearse the material as this increases the likelihood of moving that knowledge to longer-term memory. At both phases there can be a retrieval process, which involves transferring the knowing and understanding from long-term memory back into short-term working memory.^30,31^

#### Acquiring surface learning

In their meta-analysis of various interventions, Hattie *et al.* ^32^ found that many learning strategies were highly effective in enhancing reproductive performances (surface learning) for virtually all students. Surface learning includes subject matter vocabulary, the content of the lesson and knowing much more. Strategies include record keeping, summarisation, underlining and highlighting, note taking, mnemonics, outlining and transforming, organising notes, training working memory, and imagery.

#### Consolidating surface learning

Once a student has begun to develop surface knowing it is then important to encode it in a manner such that it can retrieved at later appropriate moments. This encoding involves two groups of learning strategies: the first develops storage strength (the degree to which a memory is durably established or ‘well learned’) and the second develops strategies that develop retrieval strength (the degree to which a memory is accessible at a given point in time).^33^ ‘Encoding’ strategies are aimed to develop both, but with a particular emphasis on developing retrieval strength.^34^ Both groups of strategies invoke an investment in learning, and this involves ‘the tendency to seek out, engage in, enjoy and continuously pursue opportunities for effortful cognitive activity.^35^ Although some may not ‘enjoy’ this phase, it does involve a willingness to practice, to be curious and to explore again, and a willingness to tolerate ambiguity and uncertainty during this investment phase. In turn, this requires sufficient metacognition and a calibrated sense of progress towards the desired learning outcomes. Strategies include practice testing, spaced versus mass practice, teaching test taking, interleaved practice, rehearsal, maximising effort, help seeking, time on task, reviewing records, learning how to receive feedback and deliberate practice (i.e., practice with help of an expert, or receiving feedback during practice).

#### Acquiring deep learning

Students who have high levels of awareness, control or strategic choice of multiple strategies are often referred to as ‘self-regulated’ or having high levels of metacognition. In *Visible Learning*, Hattie^36^ described these self-regulated students as ‘becoming like teachers’, as they had a repertoire of strategies to apply when their current strategy was not working, and they had clear conceptions of what success on the task looked like.^37^ More technically, Pintrich *et al.* ^38^ described self-regulation as ‘an active, constructive process whereby learners set goals for their learning and then attempt to monitor, regulate and control their cognition, motivation and behaviour, guided and constrained by their goals and the contextual features in the environment’. These students know the what, where, who, when and why of learning, and the how, when and why to use which learning strategies.^39^ They know what to do when they do not know what to do. Self-regulation strategies include elaboration and organisation, strategy monitoring, concept mapping, metacognitive strategies, self-regulation and elaborative interrogation.

#### Consolidating deep learning

Once a student has acquired surface and deep learning to the extent that it becomes part of their repertoire of skills and strategies, we may claim that they have ‘automatised’ such learning—and in many senses this automatisation becomes an ‘idea’, and so the cycle continues from surface idea to deeper knowing that then becomes a surface idea, and so on.^40^ There is a series of learning strategies that develop the learner’s proficiency to consolidate deeper thinking and to be more strategic about learning. These include self-verbalisation, self-questioning, self-monitoring, self-explanation, self-verbalising the steps in a problem, seeking help from peers and peer tutoring, collaborative learning, evaluation and reflection, problem solving and critical thinking techniques.

#### Transfer

There are skills involved in transferring knowledge and understanding from one situation to a new situation. Indeed, some have considered that successful transfer could be thought as synonymous with learning.^41,42^ There are many distinctions relating to transfer: near and far transfer,^43^ low and high transfer,^44^ transfer to new situations and problem solving transfer,^5^ and positive and negative transfer.^45^ Transfer is a dynamic, not static, process that requires learners to actively choose and evaluate strategies, consider resources and surface information, and, when available, to receive or seek feedback to enhance these adaptive skills. Reciprocal teaching is one program specifically aiming to teach these skills; for example, Bereiter and Scardamalia^46^ have developed programs in the teaching of transfer in writing, where students are taught to identify goals, improve and elaborate existing ideas, strive for idea cohesion, present their ideas to groups and think aloud about how they might proceed. Similarly, Schoenfeld^47^ outlined a problem-solving approach to mathematics that involves the transfer of skills and knowledge from one situation to another. Marton^48^ argued that transfer occurs when the learner learns strategies that apply in a certain situation such that they are enabled to do the same thing in another situation when they realise that the second situation resembles (or is perceived to resemble) the first situation. He claimed that not only sameness, similarity, or identity might connect situations to each other, but also small differences might connect them as well. Learning how to detect such differences is critical for the transfer of learning. As Heraclitus claimed, no two experiences are identical; you do not step into the same river twice.

### Overall messages from the model

There are four main messages to be taken from the model. First, if the success criteria is the retention of accurate detail (surface learning) then lower-level learning strategies will be more effective than higher-level strategies. However, if the intention is to help students understand context (deeper learning) with a view to applying it in a new context (transfer), then higher level strategies are also needed. An explicit assumption is that higher level thinking requires a sufficient corpus of lower level surface knowledge to be effective—one cannot move straight to higher level thinking (e.g., problem solving and creative thought) without sufficient level of content knowledge. Second, the model proposes that when students are made aware of the nature of success for the task, they are more likely to be more involved in investing in the strategies to attain this target. Third, transfer is a major outcome of learning and is more likely to occur if students are taught how to detect similarities and differences between one situation and a new situation before they try to transfer their learning to the new situation. Hence, not one strategy may necessarily be best for all purposes. Fourth, the model also suggests that students can be advantaged when strategy training is taught with an understanding of the conditions under which the strategy best works—when and under what circumstance it is most appropriate.

## The current study

The current study synthesises the many studies that have related various learning strategies to outcomes. This study only pertains to achievement outcomes (skill, on the model of learning); further work is needed to identify the strategies that optimise the dispositions (will) and the motivation (thrill) outcomes. The studies synthesised here are from four sources. First, there are the meta-analyses among the 1,200 meta-analyses in *Visible Learning* that relate to strategies for learning.^36,49,50^ Second, there is the meta-analysis conducted by Lavery^51^ on 223 effect-sizes derived from 31 studies relating to self-regulated learning interventions. The third source is two major meta-analyses by a Dutch team of various learning strategies, especially self-regulation. And the fourth is a meta-analysis conducted by Donoghue *et al.* ^52^ based on a previous analysis by Dunlosky *et al.* ^53^

The data in *Visible Learning* is based on 800 meta-analyses relating influences from the home, school, teacher, curriculum and teaching methods to academic achievement. Since its publication in 2009, the number of meta-analyses now exceeds 1,200, and those influences specific to learning strategies are retained in the present study. Lavery^51^ identified 14 different learning strategies and the overall effect was 0.46—with greater effects for organising and transforming (i.e., deliberate rearrangement of instructional materials to improve learning, *d*=0.85) and self-consequences (i.e., student expectation of rewards or punishment for success or failure, *d*=0.70). The lowest effects were for imagery (i.e., creating or recalling vivid mental images to assist learning, *d*=0.44) and environmental restructuring (i.e., efforts to select or arrange the physical setting to make learning easier, *d*=0.22). She concluded that the higher effects involved ‘teaching techniques’ and related to more ‘deep learning strategies’, such as organising and transforming, self-consequences, self-instruction, self-evaluation, help-seeking, keeping records, rehearsing/memorising, reviewing and goal-setting. The lower ranked strategies were more ‘surface learning strategies’, such as time management and environmental restructuring.

Of the two meta-analyses conducted by the Dutch team, the first study, by Dignath*et al.* ^9^ analysed 357 effects from 74 studies (*N*=8,619). They found an overall effect of 0.73 from teaching methods of self-regulation. The effects were large for achievement (elementary school, 0.68; high school, 0.71), mathematics (0.96, 1.21), reading and writing (0.44, 0.55), strategy use (0.72, 0.79) and motivation (0.75, 0.92). In the second study, Donker *et al.* ^54^ reviewed 180 effects from 58 studies relating to self-regulation training, reporting an overall effect of 0.73 in science, 0.66 in mathematics and 0.36 in reading comprehension. The most effective strategies were cognitive strategies (rehearsal 1.39, organisation 0.81 and elaboration 0.75), metacognitive strategies (planning 0.80, monitoring 0.71 and evaluation 0.75) and management strategies (effort 0.77, peer tutoring 0.83, environment 0.59 and metacognitive knowledge 0.97). Performance was almost always improved by a combination of strategies, as was metacognitive knowledge. This led to their conclusion that students should not only be taught which strategies to use and how to apply them (declarative knowledge or factual knowledge) but also when (procedural or how to use the strategies) and why to use them (conditional knowledge or knowing when to use a strategy).

Donoghue *et al.* ^52^ conducted a meta-analysis based on the articles referenced in Dunlosky *et al.* ^53^ They reviewed 10 learning strategies and a feature of their review is a careful analysis of possible moderators to the conclusions about the effectiveness of these learning strategies, such as learning conditions (e.g., study alone or in groups), student characteristics (e.g., age, ability), materials (e.g., simple concepts to problem-based analyses) and criterion tasks (different outcome measures).

In the current study, we independently assigned all strategies to the various parts of the model—this was a straightforward process, and the few minor disagreements were resolved by mutual agreement. All results are presented in Appendix 1.

## Results: the meta-synthesis of learning strategies

There are 302 effects derived from the 228 meta-analyses from the above four sources that have related some form of learning strategy to an achievement outcome. Most are experimental–control studies or pre–post studies, whereas some are correlations (*N*=37). There are 18,956 studies (although some may overlap across meta-analyses). Only 125 meta-analyses reported the sample size (*N*=11,006,839), but if the average (excluding the outlier 7 million from one meta-analysis) is used for the missing sample sizes, the best estimate of sample size is between 13 and 20 million students.

**Figure 2:**
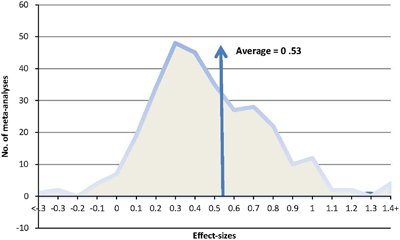
The average and the distribution of all effect sizes.

The average effect is 0.53 but there is considerable variance (Figure 2), and the overall number of meta-analyses, studies, number of people (where provided), effects and average effect-sizes for the various phases of the model are provided in Table 1. The effects are lowest for management of the environment and ‘thrill’ (motivation), and highest for developing success criteria across the learning phases. The variance is sufficiently large, however, that it is important to look at specific strategies within each phase of the model.

### Synthesis of the input phases of the model

#### The inputs: skills

There are nine meta-analyses that have investigated the relation between prior achievement and subsequent achievement, and not surprisingly these relations are high (Table 2). The average effect-size is 0.77 (s.e.=0.10), which translates to a correlation of 0.36—substantial for any single variable. The effects of prior achievement are lowest in the early years, and highest from high school to university. One of the purposes of school, however, is to identify those students who are underperforming relative to their abilities and thus to not merely accept prior achievement as destiny. The other important skill is working memory—which relates to the amount of information that can be retained in short-term working memory when engaged in processing, learning, comprehension, problem solving or goal-directed thinking.^55^ Working memory is strongly related to a person’s ability to reason with novel information (i.e., general fluid intelligence.^56^

#### The inputs: will

There are 28 meta-analyses related to the dispositions of learning from 1,304 studies and the average effect-size is 0.48 (s.e.=0.09; Table 3). The effect of self-efficacy is highest (*d*=0.90), followed by increasing the perceived value of the task (*d*= 0.46), reducing anxiety (*d*=0.45) and enhancing the attitude to the content (*d*=0.35). Teachers could profitably increase students’ levels of confidence and efficacy to tackle difficult problems; not only does this increase the probability of subsequent learning but it can also help reduce students’ levels of anxiety. It is worth noting the major movement in the anxiety and stress literature in the 1980s moved from a preoccupation on understanding levels of stress to providing coping strategies—and these strategies were powerful mediators in whether people coped or not.^57^ Similarly in learning, it is less the levels of anxiety and stress but the development of coping strategies to deal with anxiety and stress. These strategies include being taught to effectively regulate negative emotions;^58^ increasing self-efficacy, which relates to developing the students conviction in their own competence to attain desired outcomes;^59^ focusing on the positive skills already developed; increasing social support and help seeking; reducing self-blame; and learning to cope with error and making mistakes.^60^ Increasing coping strategies to deal with anxiety and promoting confidence to tackle difficult and challenging learning tasks frees up essential cognitive resources required for the academic work.

There has been much discussion about students having growth—or incremental—mindsets (human attributes are malleable not fixed) rather than fixed mindsets (attributes are fixed and invariant).^61^ However, the evidence in Table 3 (*d*=0.19) shows how difficult it is to change to growth mindsets, which should not be surprising as many students work in a world of schools dominated by fixed notions—high achievement, ability groups, and peer comparison.

#### The inputs: thrill

The thrill relates to the motivation for learning: what is the purpose or approach to learning that the student adopts? Having a surface or performance approach motivation (learning to merely pass tests or for short-term gains) or mastery goals is not conducive to maximising learning, whereas having a deep or achieving approach or motivation is helpful (Table 4). A possible reason why mastery goals are not successful is that too often the outcomes of tasks and assessments are at the surface level and having mastery goals with no strategic sense of when to maximise them can be counter-productive.^62^ Having goals, *per se*, is worthwhile—and this relates back to the general principle of having notions of what success looks like before investing in the learning. The first step is to teach students to have goals relating to their upcoming work, preferably the appropriate mix of achieving and deep goals, ensure the goals are appropriately challenging and then encourage students to have specific intentions to achieve these goals. Teaching students that success can then be attributed to their effort and investment can help cement this power of goal setting, alongside deliberate teaching.

#### The environment

Despite the inordinate attention, particularly by parents, on structuring the environment as a precondition for effective study, such effects are generally relatively small (Table 5). It seems to make no differences if there is background music, a sense of control over learning, the time of day to study, the degree of social support or the use of exercise. Given that most students receive sufficient sleep and exercise, it is perhaps not surprising that these are low effects; of course, extreme sleep or food deprivation may have marked effects.

#### Knowing the success criteria

A prediction from the model of learning is that when students learn how to gain an overall picture of what is to be learnt, have an understanding of the success criteria for the lessons to come and are somewhat clear at the outset about what it means to master the lessons, then their subsequent learning is maximised. The overall effect across the 31 meta-analyses is 0.54, with the greatest effects relating to providing students with success criteria, planning and prediction, having intentions to implement goals, setting standards for self-judgements and the difficulty of goals (Table 6). All these learning strategies allow students to see the ‘whole’ or the gestalt of what is targeted to learn before starting the series of lessons. It thus provides a ‘coat hanger’ on which surface-level knowledge can be organised. When a teacher provides students with a concept map, for example, the effect on student learning is very low; but in contrast, when teachers work together with students to develop a concept map, the effect is much higher. It is the working with students to develop the main ideas, and to show the relations between these ideas to allow students to see higher-order notions, that influences learning. Thus, when students begin learning of the ideas, they can begin to know how these ideas relate to each other, how the ideas are meant to form higher order notions, and how they can begin to have some control or self-regulation on the relation between the ideas.

### Synthesis of the learning phases of the model

#### Acquiring surface learning

There are many strategies, such as organising, summarising, underlining, note taking and mnemonics that can help students master the surface knowledge (Table 7). These strategies can be deliberately taught, and indeed may be the only set of strategies that can be taught irrespective of the content. However, it may be that for some of these strategies, the impact is likely to be higher if they are taught within each content domain, as some of the skills (such as highlighting, note taking and summarising) may require specific ideas germane to the content being studied.

While it appears that training working memory can have reasonable effects (*d*=0.53) there is less evidence that training working memory transfers into substantial gains in academic attainment.^63^ There are many emerging and popular computer games that aim to increase working memory. For example, CogMed is a computer set of adaptive routines that is intended to be used 30–40 min a day for 25 days. A recent meta-analysis (by the commercial owners^64^) found average effect-sizes (across 43 studies) exceed 0.70, but in a separate meta-analysis of 21 studies on the longer term effects of CogMed, there was zero evidence of transfer to subjects such as mathematics or reading^65^. Although there were large effects in the short term, they found that these gains were not maintained at follow up (about 9 months later) and no evidence to support the claim that working memory training produces generalised gains to the other skills that have been investigated (verbal ability, word decoding or arithmetic) even when assessment takes place immediately after training. For the most robust studies, the effect of transfer is zero. It may be better to reduce working memory demands in the classroom.^66^

#### Consolidating surface learning

The investment of effort and deliberate practice is critical at this consolidation phase, as are the abilities to listen, seek and interpret the feedback that is provided (Table 8). At this consolidation phase, the task is to review and practice (or overlearn) the material. Such investment is more valuable if it is spaced over time rather than massed. Rehearsal and memorisation is valuable—but note that memorisation is not so worthwhile at the acquisition phase. The difficult task is to make this investment in learning worthwhile, to make adjustments to the rehearsal as it progresses in light of high levels of feedback, and not engage in drill and practice. These strategies relating to consolidating learning are heavily dependent on the student’s proficiency to invest time on task wisely,^67^ to practice and learn from this practice and to overlearn such that the learning is more readily available in working memory for the deeper understanding.

#### Acquiring deeper learning

Nearly all the strategies at this phase are powerful in enhancing learning (Table 9). The ability to elaborate and organise, monitor the uses of the learning strategies, and have a variety of metacognitive strategies are the critical determinants of success at this phase of learning. A major purpose is for the student to deliberately activate prior knowledge and then make relations and extensions beyond what they have learned at the surface phase.

#### Consolidating deep learning

At this phase, the power of working with others is most apparent (Table 10). This involves skills in seeking help from others, listening to others in discussion and developing strategies to ‘speak’ the language of learning. It is through such listening and speaking about their learning that students and teachers realise what they do deeply know, what they do not know and where they are struggling to find relations and extensions. An important strategy is when students become teachers of others and learn from peers, as this involves high levels of regulation, monitoring, anticipation and listening to their impact on the learner.

There has been much research confirming that teaching help-seeking strategies is successful, but how this strategy then works in classrooms is more complex. Teachers have to welcome students seeking help, and there needs to be knowledgeable others (e.g., peers) from whom to seek the help—too often students left in unsupported environments can seek and gain incorrect help and not know the help is incorrect.^68^ Ryan and Shin^69^ also distinguished between adaptive help seeking (seeking help from others, such as an explanation, a hint, or an example, that would further learning and promote independent problem solving in the future) and expedient help seeking (seeking help that expedites task completion, such as help that provides the answer and is not focused on learning). They showed that adaptive help seeking from peers declines and expedient help seeking increases during early adolescence. Further, increases in expedient help seeking were associated with declines in achievement but changes in adaptive help seeking were unrelated to achievement. The key is for teachers to teach adaptive help seeking, to ensure the help is dependable and correct and to see this more of a student than a teacher skill. Help seeking needs to be welcomed before it can have an effect.

#### Transfer

The transfer model promoted by Marton^48^ seems to be supported in that a key in teaching for transfer involves understanding the patterns, similarities and differences in the transfer before applying the strategies to new task (Table 11). Marton argued that transfer occurs when students learn strategies that apply in a certain situation such that they are enabled to do the same thing in another situation to the degree that they realise how the second situation does (or does not) resemble the first situation. It is learning to detect differences and similarities that is the key that leads to transfer of learning.

## Discussion and Conclusions

There is much debate about the optimal strategies of learning, and indeed we identified >400 terms used to describe these strategies. Our initial aim was to rank the various strategies in terms of their effectiveness but this soon was abandoned. There was too much variability in the effectiveness of most strategies depending on when they were used during the learning process, and thus we developed the model of learning presented in this article. Like all models, it is a conjecture, it aims to say much and it is falsifiable. The efficacy of any model can be seen as an expression of its capacity to generate a scalable solution to a problem or need in ways that resolve more issues than prevailing theories or approaches.^70^ The model posits that learning must be embedded in some content (something worth knowing) and thus the current claims about developing 21st century skills *sui generis* are most misleading. These skills often are promoted as content free and are able to be developed in separate courses (e.g., critical thinking, resilience). Our model, however, suggests that such skills are likely to be best developed relative to some content. There is no need to develop learning strategy courses, or teach the various strategies outside the context of the content. Instead, the strategies should be an integral part of the teaching and learning process, and can be taught within this process.

The model includes three major inputs and outcomes. These relate to what the students bring to the learning encounter (skill), their dispositions about learning (will) and their motivations towards the task (thrill). The first set of strategies relate to teaching students the standards for what is to be learned (the success criteria). We propose that effective learning strategies will be different depending on the phase of the learning—the strategies will be different when a student is first acquiring the matters to be learnt compared with when the student is embedding or consolidating this learning. That is, the strategies are differentially effective depending on whether the learning intention is surface learning (the content), deep learning (the relations between content) or the transfer of the skills to new situations or tasks. In many ways this demarcation is arbitrary (but not capricious) and more experimental research is needed to explore these conjectures. Further, the model is presented as linear whereas there is often much overlap in the various phases. For example, to learn subject matter (surface) deeply (i.e., to encode in memory) is helped by exploring and understanding its meaning; success criteria can have a mix of surface and deep and even demonstrate the transfer to other (real world) situations; and often deep learning necessitates returning to acquire specific surface level vocabulary and understanding. In some cases, there can be multiple overlapping processes. A reviewer provided a clear example: in learning that the internal angles of a quadrilateral add up to 360°, this might involve surface learning, which then requires rehearsal to consolidate, some self-questioning to apply, some detection of similarities to then work out what the internal angles of a hexagon might be, and spotting similarities to the triangle rule. There may be no easy way to know the right moment, or no easy demarcation of the various phases. The proposal in this paper is but a ‘model’ to help clarify the various phases of learning, and in many real world situations there can be considerable overlap.

We have derived six sets of propositions from our conceptual model of learning and the results of our meta-synthesis of research on learning strategies. The first set relates to the differential role played by what students bring to and take from the learning encounter—the inputs and outcomes. Second, there are some strategies that are more effective than others—but their relative effectiveness depends on the phase in the model of learning in which they take place. Third is the distinction between surface learning, deep learning and the transfer of learning. The fourth set relates to the skills of transfer, the fifth to how the model of learning can be used to resolve some unexpected findings about the effectiveness of some strategies, and the sixth set discusses the question ‘what is learning?’.

### The intertwining role of skill, will, and thrill

Our first set of claims relates to the differential role of what students bring to and take from the learning encounter. Rather than arguing that many factors contribute to achievement (an important but sometimes the only privileged outcome of learning), we are promoting the notion that the skill, will and thrill can intertwine during learning and that these three inputs are also important outcomes of learning—the aim is to enhance the will (e.g., the willingness to reinvest in more and deeper learning), the thrill (e.g., the emotions associated with successful learning, the curiosity and the willingness to explore what one does not know) and the skills (e.g., the content and the deeper understanding). The relation between the thrill, will and skill can vary depending on the student and the requirements of the task. Certainly, negative emotions, such as those induced by fear, anxiety, and stress can directly and negatively affect learning and memory. Such negative emotions block learning: ‘If the student is faced with sources of stress in an educational context which go beyond the positive challenge threshold—for instance, aggressive teachers, bullying students or incomprehensible learning materials whether books or computers—it triggers fear and cognitive function is negatively affected.^71^ Our argument is that learning can lead to enhanced skills, dispositions, motivations and excitements that can be reinvested in learning, and can lead to students setting higher standards for their success criteria. When skill, will, and thrill overlap, this should be considered a bonus; developing each is a worthwhile outcome of schooling in its own right.

### It is all in the timing

Our second set of claims is that while it is possible to nominate the top 10 learning strategies the more critical conclusion is that the optimal strategies depend on where in the learning cycle the student is located. This strategic skill in using the strategies at the right moment is akin to the message in the Kenny Rogers song—you need to ‘know when to hold ‘em, know when to fold ‘em’. For example, when starting a teaching sequence, it is most important to be concerned that students have confidence they can understand the lessons, see value in the lessons and are not overly anxious about their skills to be mastered. Providing them early on with an overview of what successful learning in the lessons will look like (knowing the success criteria) will help them reduce their anxiety, increase their motivation, and build both surface and deeper understandings.

To acquire surface learning, it is worthwhile knowing how to summarise, outline and relate the learning to prior achievement; and then to consolidate this learning by engaging in deliberate practice, rehearsing over time and learning how to seek and receive feedback to modify this effort. To acquire deep understanding requires the strategies of planning and evaluation and learning to monitor the use of one’s learning strategies; and then to consolidate deep understanding calls on the strategy of self-talk, self-evaluation and self-questioning and seeking help from peers. Such consolidation requires the learner to think aloud, learn the ‘language of thinking’,^72^ know how to seek help, self-question and work through the consequences of the next steps in learning. To transfer learning to new situations involves knowing how to detect similarities and differences between the old and the new problem or situations.

We recommend that these strategies are developed by embedding them into the cycle of teaching rather than by running separate sessions, such as ‘how to learn’ or study skills courses. There is a disappointing history of educational programs aimed at teaching students how to learn.^30,73,74^ Wiliam^75^ made this case for why teaching these learning strategies (e.g., critical thinking) out of context is unlikely to develop a generic skill applicable to many subjects. He noted that in a ‘mathematics proof, critical thinking might involve ensuring that each step follows from the previous one (e.g., by checking that there has not been a division by zero). In reading a historical account, critical thinking might involve considering the author of the account, the potential biases and limitations that the author may be bringing to the account, and what other knowledge the reader has about the events being described. The important point here is that although there is some commonality between the processes in mathematics and history, they are not the same. Developing a capacity for critical thinking in history does not make one better at critical thinking in mathematics. For all of the apparent similarities, critical thinking in history and critical thinking in mathematics are different, and they are developed in different ways’. Many others have noted that metacognition is not knowledge-free but needs to be taught in the context of the individual subject areas.^76,77^ Perkins^78^ also noted that there is a certain art to infusing the teaching of thinking into content learning. Sometimes, ‘teachers think it is enough simply to establish a generally thoughtful atmosphere in a classroom, with regular expectations for thinking critically and creatively...teaching for know-how about learning to learn is a much more time-consuming enterprise than teaching for just learning the ideas... Building active know-how requires much more attention’.

Another aspect to consider is the difference, identified in the model, between being first exposed to learning and the consolidation of this learning. This distinction is far from novel. Shuell,^79^ for example, distinguished between initial, intermediate, and final phases of learning. In the initial phase, the students can encounter a ‘large array of facts and pieces of information that are more-or-less isolated conceptually... there appears to be little more than a wasteland with few landmarks to guide the traveller on his or her journey towards understanding and mastery’. Students can use existing schema to make sense of this new information, or can be guided to have more appropriate schema (and thus experience early stages of concept learning and relation between ideas) otherwise the information may remain as isolated facts, or be linked erroneously to previous understandings. At the intermediate phase, the learner begins to see similarities and relationships among these seemingly conceptually isolated pieces of information. ‘The fog continues to lift but still has not burnt off completely’. During the final phase, the knowledge structure becomes well integrated and functions more autonomously, and the emphasis is more on performance or exhibiting the outcome of learning.

### Horses for courses: matching strategies with phases

The third set of claims relates to the distinction between surface, deep, and transfer of learning. Although not a hard and fast set of demarcations, surface learning refers more to the content and underlying skills; deep learning to the relationships between, and extensions of, ideas; and transfer to the proficiency to apply learning to new problems and situations. During the surface learning phase, an aim is to assist students to overlearn certain ideas and thus reduce the needs of their working memory to work with these new facts when moving into the deeper understanding phase. Note, for example, that Marton *et al.* ^80^ made an important distinction between memorising without understanding first and called this rote memorisation (which has long term effect), and memorisation when you have understood and called this meaningful memorisation (which can be powerful). The evidence in the current study supports this distinction.

It is when students have much information, or many seemingly unrelated ideas, that the learning strategies for the deep phase are optimally invoked. This is when they should be asked to integrate ideas with previous schema or modify their previous schema to integrate new ideas and ways of thinking. The key to this process is first gaining ideas—a fact often missed by those advocating deeper thinking strategies when they try to teach these skills prior to developing sufficient knowledge within the content domain. The students need to first have ideas before they can relate them. The model does not propose discarding the teaching or learning skills that have been developed to learn surface knowing, but advocates the benefits of a more appropriate balance of surface and deeper strategies and skills that then lead to transfer. The correct balance of surface to deep learning depends on the demands of the task. It is likely that more emphasis on surface strategies is probably needed as students learn new ideas, moving to an emphasis on deeper strategies as they become more proficient.

### Pause and reflect: detecting similarities and differences

The fourth set of claims relate to the skills of transfer, and how important it is to teach students to pause and detect the similarities and differences between previous tasks and the new one, before attempting to answer a new problem. Such transfer can be positive, such as when a learner accurately remembers a learning outcome reached in a certain situation and appropriately applies it in a new and similar situation, or negative, such as when a learner applies a strategy used successfully in one situation in a new situation where this strategy is not appropriate. Too many (particularly struggling) students over-rehearse a few learning strategies (e.g., copying and highlighting) and apply them in situations regardless of the demands of new tasks. Certainly, the fundamental skill for positive transfer is stopping before addressing the problem and asking about the differences and similarities of the new to any older task situation. This skill can be taught.

This ability to notice similarities and differences over content is quite different for novices and experts^81,82^ and we do not simply learn from experience but we also learn to experience.^83^ Preparation for future learning involves opportunities to try our hunches in different contexts, receive feedback, engage in productive failure and learn to revise our knowing based on feedback. The aim is to solve problems more efficiently, and also to ‘let go’ of previously acquired knowledge in light of more sophisticated understandings—and this can have emotional consequences: ‘Failure to change strategies in new situations has been described as the tyranny of success’.^84^ It is not always productive for students to try the same thing that worked last time. Hence there may need to be an emphasis on knowledge-building rather than knowledge-telling,^85^ and systematic inquiry based on theory-building and disconfirmation rather than simply following processes for how to find some result.

### Why some strategies do not work

The fifth set of claims relate to how the model can be used to resolve some of the unexpected findings about the impact of various teaching methods. In *Visible Learning*,^36^ it was noted that many programs that seem to lead to developing deeper processing have very low effect sizes (e.g., inquiry based methods, *d*=0.31; problem-based learning, *d*=0.15). For example, there have been 11 meta-analyses relating to problem-based learning based on 509 studies, leading to an average small effect (*d*=0.15). It hardly seems necessary to run another problem-based program (particularly in first-year medicine, where four of the meta-analyses were completed) to know that the effects of problem-based learning on outcomes are small. The reason for this low effect seems to be related to using problem-based methods before attaining sufficient surface knowledge. When problem-based learning is used in later medical years, the effects seem to increase. Albanese and Mitchell^86^ claimed that increased years of exposure to medical education increases the effect of problem-based learning. They argued that lack of experience (and lack of essential surface knowledge) leads the student to make more errors in their knowledge base, add irrelevant material to their explanations and engage in backward reasoning (from the unknown to the givens), whereas experts engaged in forward reasoning (also see references 87,^88^). Walker *et al.* ^89^ also noted that novice problem-based learning students tended to engage in far more backward-driven reasoning, which results in more errors during problem solving and may persist even after the educational intervention is complete. It is likely that problem-based learning works more successfully when students engage in forward reasoning and this depends on having sufficient content knowledge to make connections.

Deep understanding in problem-based learning requires a differentiated knowledge structure,^90^ and this may need to be explicitly taught—as there is no assumption that students will see similarities and differences in contexts by themselves. There is a limit to what we can reasonably expect students to discover, and it may require teaching students to make predictions based on features that were told to them and that they may not notice on their own. Deliberate teaching of these surface features can offer a higher level of explanation that would be difficult or time consuming to discover. A higher level explanation is important because it provides a generative framework that can extend one understanding beyond the specific cases that have been analysed and experienced. On the other hand, the problems need not be too overly structured, as then students do not gain experience of searching out conceptual tools or homing in on particular cases of application.^78^

Another example of the different requirements of surface and deep learning is the effect of asking students to explore errors and misconceptions during their learning. Using meta-analysis, Keith and Frese^91^ found that the average effect of using these strategies when the outcome was surface learning was −0.15 and when the outcome was deep learning and far transfer to new problems, it was 0.80.

### So: what is learning?

The sixth set of claims relate to the notion of ‘what is learning?’. The argument in this article is that learning is the outcome of the processes of moving from surface to deep to transfer. Only then will students be able to go beyond the information given to ‘figure things out’, which is one of the few untarnishable joys of life.^92^ One of the greatest triumphs of learning is what Perkins^78^ calls ‘knowing one’s way around’ a particular topic or ‘playing the whole game’ of history, mathematics, science or whatever. This is a function of knowing much and then using this knowledge in the exploration of relations and to make extensions to other ideas, and being able to know what to do when one does not know what to do (the act of transfer).

### Concluding comments

Like all models, the one proposed in this article invites as many conjectures and directions for further research as it provide a basis for interpreting the evidence from the meta-synthesis. It helps make sense of much of the current literature but it is speculative in that it also makes some untested predictions. There is much solace in Popper's^93^ claim that ‘Bold ideas, unjustified anticipations, and speculative thought, are our only means for interpreting nature: our only organon, our only instrument, for grasping her. And we must hazard them to win our prize. Those among us who are unwilling to expose their ideas to the hazard of refutation do not take part in the scientific game.’ Further research is needed, for example, to better understand the optimal order through the various phases; there may be circumstances where it may be beneficial to learn the deeper notions before developing the surface knowledge. It is highly likely that as one develops many ideas and even relates and extends them, these become ‘ideas’ and the cycle continues.^94^ We know much, but we need to know much more, and in particular we need to know how these many learning strategies might be better presented in another competing model. Such testing of a bold model and making predictions from models is, according to Popper, how science progresses.

Further research is needed that asks whether the distinction between the acquisition and the consolidation of learning is a distinctive difference, a melding from one to the other or whether both can occur simultaneously. If there is a difference, then more research on ascertaining the best time to move from acquisition to consolidation would be informative. Similarly, there is no hard rule in the model of a sequence from surface to deep to transfer. In some ways, teaching the strategies of knowing what success looks like upfront implies an exposure to both surface and deep learning. Also, the many arguments (but surprisingly there is a lack of evidence) for the popular notions of flipped classrooms could be supported with more evidence of introducing the success criteria upfront to students. A typical flipped lesson starts with students accessing online video lectures or resources prior to in-class sessions so that students are prepared to participate in more interactive and higher-order activities such as problem solving, discussions and debates.^95^ The most needed research concerns transfer—the variation theory of Marton,^48^ the claims by Perkins^78^ and others need more focused attention and the usual (and often unsubstantiated) claims that doing *x* will assist learning *y* should come back as a focus of learning sciences.

We are proposing that it is worthwhile to develop the skill, will and thrill of learning, and that there are many powerful strategies for learning. Students can be taught these strategies (declarative knowledge), how to use them (procedural knowledge), under what conditions it may be more or less useful to apply them (conditional knowledge) and how to evaluate them. It may be necessary to teach when best to use these strategies according the nature of the outcomes (surface and deep), according to the timing of learning (first acquiring and then consolidating learning) and to teach the skill of transferring learning to new situations. We need to think in terms of ‘surface to deep’ and not one alone; we need to think in terms of developing dispositions, motivations and achievement, and not one alone. This invites considering multiple outcomes from our schools. Singapore,^96^ for example, is now committed to developing an educational system which will produce young people who have the moral courage to stand up for what is right; pursue a healthy lifestyle and have an appreciation of aesthetics; are proud to be Singaporeans; are resilient in the face of difficulty, innovative and enterprising; are purposeful in the pursuit of excellence; are able to collaborate across cultures; and can think critically and communicate persuasively. Academic achievement is but one desirable learning outcomes of many.

Another important message is that developing a few learning strategies may not be optimal. The failure to change strategies in new situations has been described as the tyranny of success;^84^ and the current meta-synthesis suggests that choosing different strategies as one progresses through the learning cycle (from first exposure to embedding, from surface to deep to transfer) demands cognitive flexibility. It may not be the best option for students to use the same strategies that worked last time, as when the context is changed the old strategies may no longer work.

## Figures and Tables

**Table 1:** Overall summary statistics for the learning strategies synthesis

	*No. of metas*	*No. of studies*	*No. of people*	*Prorated people*	*No. of effects*	*ES*
Skill	13	3,371	136,270	229,370	9,572	0.75
Will	28	1,304	1,468,335	1,601,335	5,081	0.48
Thrill–motivation	23	1,468	451,899	638,099	4,478	0.34
Managing the environment	24	1,056	157,712	330,612	3,928	0.17
Success criteria	41	3,395	57,850	416,950	5,176	0.55
Acquiring surface learning	26	935	26,656	226,156	2,156	0.63
Consolidating surface learning	71	3,366	7,296,722	7,921,822	6,216	0.57
Acquiring deep learning	14	1,066	1,314,618	1,367,818	2,582	0.57
Consolidating deep learning	58	2,885	96,776	602,176	7,196	0.53
Transfer	3	110		39,900	173	1.09
Total	301	18,956	11,006,839	13,374,239	46,558	0.53

**Table 2:** Meta-analysis results for ‘the skill’

*Skill*	*No. of metas*	*No. of studies*	*No. of people*	*Prorated No. of people*	*No. of effects*	*ES*
Prior achievement	9	3,155	113,814	193,614	8,014	0.77
Working memory	4	216	22,456	35,756	1,558	0.68

**Table 3:** Meta-analysis results for ‘the will’

*Will*	*No. of metas*	*No. of studies*	*No. of people*	*Prorated No. of people*	*No. of effects*	*ES*
Self-efficacy	5	140	27,062	53,662	143	0.90
Task value	1	6		13,300	6	0.46
Reducing anxiety	8	247	105,370	158,570	1,305	0.45
Self-concept	6	440	345,455	372,055	2,548	0.41
Attitude to content	4	320	957,609	970,909	782	0.35
Mindfulness	3	66	4,622	4,622	184	0.29
Incremental versus entity thinking	1	85	28,217	28,217	113	0.19

**Table 4:** Meta-analysis results for ‘the thrill’

*Thrill*–*motivation*	*No. of metas*	*No. of studies*	*No. of people*	*Prorated No. of people*	*No. of effects*	*ES*
Deep motivation	1	72		13,300	72	0.75
Achieving approach	1	95		13,300	95	0.70
Deep approach	1	38		13,300	38	0.63
Goals (Mastery, performance, social)	11	587	348,346	401,546	3,584	0.48
Mastery goals (general)	3	158	12,466	39,066	163	0.19
Achieving motivation	1	18		13,300	18	0.18
Surface/ performance approach	2	344	91,087	104,387	344	0.11
Surface/ performance motivation	3	156		39,900	164	−0.19

**Table 5:** Meta-analysis results for the environment

*Management of the environment*	*No. of metas*	*No. of studies*	*No. of people*	*Prorated No. of people*	*No. of effects*	*ES*
Environmental structuring	2	10		26,600	10	0.41
Time management	2	86		26,600	86	0.40
Exercise	8	397	30,206	96,706	2,344	0.26
Social support	1	33	12,366	12,366	33	0.12
Time of day to study	3	267	31,229	44,529	1,155	0.12
Student control over learning	4	124	7,993	34,593	161	0.02
Background music	1	43	3,104	3,104	43	−0.04
Sleep	3	96	72,814	86,114	96	−0.05

**Table 6:** Meta-analysis results for success criteria

*Knowing success criteria*	*No. of metas*	*No. of studies*	*No. of people*	*Prorated No. of people*	*No. of effects*	*ES*
Success criteria	1	7		13,300	7	1.13
Planning and prediction	4	399		53,200	420	0.76
Goal intentions	2	81	8,461	21,761	190	0.68
Concept mapping	9	1,049	9,279	75,779	1,141	0.64
Setting standards for self-judgement	1	156		13,300	156	0.62
Goal difficulty	7	428	30,521	57,121	526	0.57
Advanced organisers	12	935	3,905	136,905	2,291	0.42
Goal commitment	3	257	2,360	28,960	266	0.37
Worked examples	2	83	3,324	16,624	179	0.37

**Table 7:** Meta-analysis results for acquiring surface learning

*Acquiring surface learning*	*No. of metas*	*No. of studies*	*No. of people*	*Prorated No. of people*	*No. of effects*	*ES*
Strategy to integrate with prior knowledge	1	10		13,300	12	0.93
Outlining and transforming	1	89		13,300	89	0.85
Mnemonics	4	80	4,705	31,305	171	0.76
Working memory training	4	191	11,854	25,154	1,006	0.72
Summarisation	2	70	1,914	15,214	207	0.66
Organising	3	104		39,900	104	0.60
Record keeping	2	177		26,600	177	0.54
Underlining and highlighting	1	16	2,070	2,070	44	0.50
Note taking	7	186	5,122	58,322	287	0.50
Imagery	1	12	991	991	59	0.45

**Table 8:** Meta-analysis results for consolidating surface learning

*Consolidating surface learning*	*No. of metas*	*No. of studies*	*No. of people*	*Prorated No. of people*	*No. of effects*	*ES*
Deliberate practice	3	161	13,689	13,689	258	0.77
Effort	1	15		13,300	15	0.77
Rehearsal and memorisation	3	132	0	39,900	132	0.73
Giving/receiving feedback	28	1,413	75,279	288,079	2,219	0.71
Spaced versus mass practice	4	360	14,811	54,711	965	0.60
Help seeking	1	62		13,300	62	0.60
Time on task	8	254	28,034	121,134	300	0.54
Reviewing records	1	8	523	523	84	0.49
Practice testing	10	674	7,147,625	7,227,425	1,598	0.44
Teaching test taking and coaching	11	275	15,772	148,772	372	0.27
Interleaved practice	1	12	989	989	65	0.21

**Table 9:** Meta-analysis results for acquiring deep learning

*Acquiring deep learning*	*No. of metas*	*No. of studies*	*No. of people*	*Prorated No. of people*	*No. of effects*	*ES*
Elaboration and organisation	1	50		13,300	50	0.75
Strategy monitoring	1	81		13,300	81	0.71
Meta-cognitive strategies	5	355	1,203,024	1,216,324	781	0.61
Self-regulation	6	556	109,444	109,444	1,506	0.52
Elaborative interrogation	1	24	2,150	15,450	164	0.42

**Table 10:** Meta-analysis results for consolidating deep learning

*Consolidating deep learning*	*No. of metas*	*No. of studies*	*No. of people*	*Prorated No. of people*	*No. of effects*	*ES*
Seeking help from peers	1	21		13,300	21	0.83
Classroom discussion	1	42		13,300	42	0.82
Evaluation and reflection	1	54		13,300	54	0.75
Self consequences	1	75		13,300	75	0.70
Problem-solving teaching	11	683	15,235	121,635	1,820	0.68
Self-verbalisation and self-questioning	4	226	6,196	19,496	2,300	0.64
via becoming a teacher (peer tutoring)	15	839	18,193	164,493	1,272	0.54
Self-explanation	1	8	533	533	69	0.50
Self-monitoring	1	154		13,300	154	0.45
Self verbalising the steps in a problem	3	154		39,900	154	0.41
Collaborative/cooperative learning	18	512	35,921	168,921	1,074	0.38
Critical thinking techniques	1	117	20,698	20,698	161	0.34

**Table 11:** Meta-analysis results for transfer

*Transfer*	*No. of metas*	*No. of studies*	*No. of people*	*Prorated No. of people*	*No. of effects*	*ES*
Similarities and differences	1	51		13,300	51	1.32
Seeing patterns to new situations	1	6		13,300	6	1.14
Far transfer	1	53		13,300	116	0.80
