# Supplementary material for: Learning strategies: a synthesis and conceptual model
Source: NPJ Sci Learn. 2016 Aug 10;1:16013. doi: 10.1038/npjscilearn.2016.13 (PMC6380372; doi:10.1038/npjscilearn.2016.13)
Supplement: Supplementary Appendix [file npjscilearn201613-s1.doc]

Appendix 1. All meta-analysis relating to learning strategies

|  |  | **Influence** | **No. studies** | **No. people** | **No. effects** | **ES** |
| --- | --- | --- | --- | --- | --- | --- |
|  | **Skill** |  |  |  |  |  |
|  | **Prior achievement** |  |  |  |  |  |
| 1 | Duncan et al. (2007) | Pre-school to first years of schooling | 6 |  | 228 | 0.35 |
| 2 | Ernst (2001) | Early cognition and school achievement | 23 | 1,733 | 32 | 0.41 |
| 3 | Kuncel, Hezlett & Ones (2001) | High school grades to university grades | 1,753 | 82,659 | 6,589 | 0.52 |
| 4 | Kavale & Nye (1985) | Ability component in predicting special ed students | 1,077 |  | 268 | 0.68 |
| 5 | Piburn (1993) | Prior ability on science achievement | 44 |  | 186 | 0.80 |
| 6 | Trapmann, Hell, Weigand, & Schuler (2007) | High school grades to university grades | 83 |  | 83 | 0.90 |
| 7 | Schuler, Funke, & Baron-Boldt (1900) | High school grades to university grades | 63 | 29,422 | 63 | 1.02 |
| 8 | Boulanger (1981) | Ability related to science learning | 34 |  | 62 | 1.09 |
| 9 | Hattie & Hansford (1983) | Intelligence and achievement | 72 |  | 503 | 1.19 |
|  | **Working memory** |  |  |  |  |  |
| 10 | Carretti, Borellam Cornoldi, & de Beni (2009( | Working memory on achievement | 18 | 3,922 | 18 | 0.71 |
| 11 | van den Bos, van den Ven, Kroesbergen, & van Luit (2013) | Working memory on achievement | 111 | 16,921 | 1,233 | 0.70 |
|  |  |  |  |  |  |  |
|  | **Will** |  |  |  |  |  |
|  | **Self-efficacy** |  |  |  |  |  |
| 12 | Dignath et al. (2008) | Self-efficacy | 13 |  | 13 | 1.84 |
| 13 | Robbins, Lauver, Le, Davis, Langley, & Carlstrom (2004) | Academic self-efficacy | 18 | 9,598 | 18 | 0.82 |
| 14 | Holden, Moncher, Schinke, & Barker (1990) | Self-efficacy | 25 |  | 26 | 0.37 |
| 15 | Multon, Brown, & Lent (1991) | Self-efficacy | 36 | 4,998 | 38 | 0.76 |
| 16 | Carpenter (2007) | Self-efficacy | 48 | 12,466 | 48 | 0.70 |
|  | **Self-concept** |  |  |  |  |  |
| 17 | Hansford & Hattie (1980) | Self-concept | 128 | 202,823 | 1,136 | 0.41 |
| 18 | Muller, Gullung, & Bocci (1988) | Self-concept | 38 |  | 838 | 0.36 |
| 19 | Wickline (2003) | Self-concept | 41 | 48,038 | 41 | 0.35 |
| 20 | Valentine, DuBois, & Cooper (2004) | Self-concept | 56 | 50,000 | 34 | 0.32 |
|  |  | **Influence** | **No. studies** | **No. people** | **No. effects** | **ES** |
| 21 | O'Mara & Marsh (2006) | Self-concept interventions | 145 |  | 460 | 0.51 |
| 22 | Huang (2011) | Self-concept | 32 | 44,594 | 39 | 0.49 |
|  | **Reducing anxiety** |  |  |  |  |  |
| 23 | Kim et al. (2008) | Lowering text anxiety | 4 |  | 9 | 1.10 |
| 24 | Hembree (1988) | Reduced test anxiety | 46 | 28,276 | 176 | 0.22 |
| 25 | Harris (1987) | Reduced anxiety on performance | 70 |  | 70 | 0.35 |
| 26 | Seipp (1991) | Reduction of anxiety on achievement | 26 | 36,626 | 156 | 0.43 |
| 27 | Bourhis & Allen (1992) | Lack of communication apprehension | 23 |  | 728 | 0.37 |
| 28 | Taylor (1995) | Reducing anxiety in math | 25 | 22,189 | 47 | 0.31 |
| 29 | Fitzgerald (1996) | Reducing anxiety to statistics | 27 |  | 82 | 0.28 |
| 30 | Ma (1999) | Reducing anxiety towards math and achievement | 26 | 18,279 | 37 | 0.56 |
|  | **Task value** |  |  |  |  |  |
| 31 | Dignath et al. (2008) | Task value | 6 |  | 6 | 0.46 |
|  | **Attitude to content** |  |  |  |  |  |
| 32 | Willson (1983) | Attitudes to science | 43 | 638,333 | 280 | 0.32 |
| 33 | Bradford (1991) | Attitude to mathematics | 102 |  | 241 | 0.29 |
| 34 | Ma & Kishor (1997) | Attitude to mathematics | 143 | 94,661 | 143 | 0.47 |
| 35 | Petscher (2010) | Attitude to reading and achievement | 32 | 224,615 | 118 | 0.32 |
|  | **Incremental vs entity thinking** |  |  |  |  |  |
| 36 | Burnette, O’Boyle, van Epps, Pollack, & Finkel (2013) | Incremental vs entity thinking | 85 | 28,217 | 113 | 0.19 |
|  | **Mindfulness** |  |  |  |  |  |
| 37 | Zenner, Hermeben-Kurz, & Walach (2014) | Mindfullness on achievement | 24 | 1,348 | 24 | 0.41 |
| 38 | Sedlmeier, Eberth, Schwarz, Zimmerman, Haarig, Jaeger, & Kunze (2012) | Mindfullness on achievement | 22 | 1,502 | 22 | 0.23 |
| 39 | Zoogman, Goldberrg, Hoyt, & Miller (2014) | Mindfullness on achievement | 20 | 1,772 | 138 | 0.23 |
|  |  |  |  |  |  |  |
|  | **Thrill** |  |  |  |  |  |
|  | **Surface approach** |  |  |  |  |  |
| 40 | Purdie & Hattie (1999) | Surface approach | 101 |  | 101 | 0.10 |
|  |  | **Influence** | **No. studies** | **No. people** | **No. effects** | **ES** |
| 41 | Hulleman, Schrager, Bodmann, & Harackiewica (2010) | Approach goals on achievement | 243 | 91,087 | 243 | 0.12 |
|  | **Surface motivation** |  |  |  |  |  |
| 42 | Purdie & Hattie (1999) | Surface motivation | 48 |  | 48 | -0.54 |
| 43 | van Yperen, Blaga, & Postmes (2014) | Performance goals on achievement | 98 |  | 106 | 0.20 |
| 44 | Rolland (2012) | Performance goals on achievement | 10 |  | 10 | -0.22 |
|  | **Deep approach** |  |  |  |  |  |
| 45 | Purdie & Hattie (1999) | Deep approach | 38 |  | 38 | 0.63 |
|  | **Deep motivation** |  |  |  |  |  |
| 46 | Purdie & Hattie (1999) | Deep motivation | 72 |  | 72 | 0.75 |
|  | **Achieving approach** |  |  |  |  |  |
| 47 | Purdie & Hattie (1999) | Achieving approach | 95 |  | 95 | 0.70 |
|  | **Achieving motivation** |  |  |  |  |  |
| 48 | Purdie & Hattie (1999) | Achieving motivation | 18 |  | 18 | 0.18 |
|  | **Mastery goals (general)** |  |  |  |  |  |
| 49 | van Yperen, Blaga & Postmes (2014) | Mastery goals on achievement | 98 |  | 103 | 0.28 |
| 50 | Rolland (2012) | Mastery goals on achievement | 12 |  | 12 | 0.04 |
| 51 | Carpenter (2007) | Mastery goals on achievement | 48 | 12,466 | 48 | 0.24 |
|  | **Goals (mastery, performance, social)** |  |  |  |  |  |
| 52 | Robbins, Lauver, Le, Davis, Langley, & Carlstrom (2004) | Achievement goals | 34 | 17,575 | 34 | 0.65 |
| 53 | Uguroglu & Walberg (1979) | Motivation | 40 | 36,946 | 232 | 0.34 |
| 54 | Findley & Cooper (1983) | Internal locus of control | 98 | 15,285 | 275 | 0.36 |
| 55 | Whitley & Frieze (1985) | Success vs failure attributions | 25 |  | 25 | 0.56 |
| 56 | Schiefel, Krapp, & Schreyer (1995) | Interest and achievement | 21 |  | 121 | 0.65 |
| 57 | Kalechstein & Nowicki (1997) | Internal locus of control | 78 | 58,142 | 261 | 0.23 |
| 58 | Crede & Phillips (2011) | Motivation strategies | 67 | 19,900 | 2,158 | 0.24 |
| 59 | Wagner & Szamoskozi (2012) | Direct academic motivation training | 17 | 3,720 | 17 | 0.33 |
| 60 | Marzano (2000) | Goal specification | 53 |  | 53 | 0.97 |
| 61 | Cerasoli, Nicklin, & Ford (2014) | Intrinsic motivation | 125 | 196,778 | 125 | 0.47 |
| 62 | Chen, Chen, & Zhu (2013) | Motivation on physical education outcomes | 29 |  | 283 | 0.43 |
|  |  |  |  |  |  |  |
|  |  | **Influence** | **No. studies** | **No. people** | **No. effects** | **ES** |
|  | **Management of the Environment** |  |  |  |  |  |
|  | **Environmental structuring** |  | |  |  |  |
| 63 | Lavery (2008) | Environmental restructuring | 4 |  | 4 | 0.22 |
| 64 | Donker et al. (2013) | Environmental restructuring | 6 |  | 6 | 0.59 |
|  | **Time management** |  |  |  |  |  |
| 65 | Lavery (2008) | Time management | 8 |  | 8 | 0.44 |
| 66 | Kyriakides, Christoforou, & Charalambous | Time management | 78 |  | 78 | 0.35 |
|  | **Social support** |  |  |  |  |  |
| 67 | Robbins, Lauver, Le, Davis, Langley, & Carlstrom (2004) | Social support | 33 | 12,366 | 33 | 0.12 |
|  | **Student control over learning** |  |  |  |  |  |
| 68 | Niemiec, Sikorski, & Walberg (1996) | Student control over learning in CAI | 24 |  | 24 | -0.03 |
| 69 | Patall, Cooper, & Robinson (2008) | Control over learning on subsequent control | 41 |  | 14 | 0.10 |
| 70 | Karich, Bruns, & Maki (2014) | Contol over learning in CAI | 18 | 3,618 | 29 | 0.05 |
| 71 | Parsons (1992) | Control over learning in CAI | 41 | 4,375 | 94 | -0.04 |
|  | **Time of day to study** |  |  |  |  |  |
| 72 | Preckel, Lipnevich, Schneider, & Roberts (2011) | Preference for morning over afternoon for intensive study | 19 | 3,920 | 19 | 0.02 |
| 73 | Richardson, Abraham, & Bond (2012) | Preference for morning over afternoon for intensive study | 217 |  | 1,105 | 0.06 |
| 74 | Tonetti, Natale, & Randler (2015) | Preference for morning over afternoon for intensive study | 31 | 27,309 | 31 | 0.29 |
|  | **Background music** |  |  |  |  |  |
| 75 | Kampfe, Sedlmeier, & Renkewit (2010) | Background music | 43 | 3,104 | 43 | -0.04 |
|  | **Sleep** |  |  |  |  |  |
| 76 | Dewald, Meijer, Oort, Kerkhof, & Bogels (2010) | Sleep quality, duration & sleepiness | 26 | 48,360 | 26 | 0.01 |
| 77 | Astill, van der Heijden, van Ijzendoorn, & van Someren (2013) | Sleep on achievement | 52 | 24,454 | 52 | 0.12 |
| 78 | Galland, Spruyt, Dawes, McDowall, Elder, & Schaughency (2013) | Sleep on achievement | 18 |  | 18 | -0.29 |
|  | **Exercise** |  |  |  |  |  |
|  |  | **Influence** | **No. studies** | **No. people** | **No. effects** | **ES** |
| 79 | Moon, Render, & Pendley (1985) | Relaxation and achievement | 20 |  | 36 | 0.16 |
| 80 | Etnier, Salazar, Landers, Petruzzelo, Han, & Nowell (1997) | Physical fitness & exercise | 134 |  | 1,260 | 0.25 |
| 81 | Sibley & Etnier (2002) | Physical activity on achievement | 36 |  | 104 | 0.36 |
| 82 | Etnier, Nowell, Landers, & Sibley (2006) | Aerobic fitness and cognitive performance | 37 | 1,306 | 571 | 0.34 |
| 83 | Verburgh, Lonigs, Scherder, & Oosterlaan (2014) | Physical activity on executive functioning | 19 | 586 | 19 | 0.52 |
| 84 | Fedewa & Ahn (2011) | Physical activity on achievement | 59 | 28,314 | 195 | 0.32 |
| 85 | Hattie & Clinton (2012) | Physical activity on achievement | 13 |  | 80 | 0.03 |
| 86 | Chang, Labbam, Gapin, & Etnier (2012) | Physical activity on achievement | 79 |  | 79 | 0.10 |
|  |  |  |  |  |  |  |
|  |  |  |  |  |  |  |
|  | **Success Criteria** |  |  |  |  |  |
|  | **Advanced organisers** |  |  |  |  |  |
| 87 | Marzano (2000) | Advance organisers | 358 |  | 358 | 0.48 |
| 88 | Kozlow & White (1978) | Advance organisers | 77 |  | 91 | 0.89 |
| 89 | Luiten, Ames, & Ackerman (1980) | Advance organisers | 135 |  | 160 | 0.21 |
| 90 | Stone (1983) | Advance organisers | 29 |  | 112 | 0.66 |
| 91 | Lott (1983) | Advance organisers in science | 16 |  | 147 | 0.24 |
| 92 | Asencio (1984) | Behavioural objectives | 111 |  | 111 | 0.12 |
| 93 | Klauer (1984) | Intentional learning | 23 |  | 52 | 0.40 |
| 94 | Rolhelser-Bennett (1987) | Advance organisers | 12 | 1,968 | 45 | 0.80 |
| 95 | Mahar (1992) | Advance organisers | 50 |  | 50 | 0.44 |
| 96 | Catts (1992) | Incidental learning | 14 |  | 80 | -0.03 |
| 97 | Catts (1992) | Intentional learning | 90 |  | 1,065 | 0.35 |
| 98 | Preiss & Gayle (2006) | Advance organisers | 20 | 1,937 | 20 | 0.46 |
|  | **Setting standards for self-judgement** |  |  |  |  |  |
| 99 | Lavery (2008) | Setting standards for self-judgement | 156 |  | 156 | 0.62 |
|  | **Planning and prediction** |  |  |  |  |  |
| 100 | Dignath et al. (2008) | Planning and prediction | 68 |  | 68 | 0.80 |
| 101 | Marzano (2000) | Information specification/ predictions | 242 |  | 242 | 0.38 |
| 102 | Donker et al. (2013) | Planning | 68 |  | 68 | 0.80 |
|  |  | **Influence** | **No. studies** | **No. people** | **No. effects** | **ES** |
| 103 | Kim et al. (2008) | Planning | 21 |  | 42 | 1.04 |
|  | **Worked examples** |  |  |  |  |  |
| 104 | Crissman (2006) | Worked examples on achievement | 62 | 3,324 | 151 | 0.57 |
| 105 | Wittwer & Renkl (2010) | Worked examples | 21 |  | 28 | 0.16 |
|  | **Success criteria** |  |  |  |  |  |
| 106 | Marzano (1998) | Cues/brief overview of success | 7 |  | 7 | 1.13 |
|  | **Goal difficulty** |  |  |  |  |  |
| 107 | Chidester & Grigsby (1984) | Goal difficulty | 21 | 1,770 | 21 | 0.44 |
| 108 | Tubbs (1986) | Goal difficulty, specificity and feedback | 87 |  | 147 | 0.58 |
| 109 | Mento, Steel, & Karren (1987) | Goal difficulty | 70 | 7,407 | 118 | 0.58 |
| 110 | Wood, Mento, & Locke (1987) | Goal difficulty | 72 | 7,548 | 72 | 0.58 |
| 111 | Wood, Mento, & Locke (1987) | Goal specificity | 53 | 6,635 | 53 | 0.43 |
| 112 | Wright (1990) | Goal difficulty | 70 | 7,161 | 70 | 0.55 |
| 113 | Burns (2004) | Degree of challenge | 55 |  | 45 | 0.82 |
|  | **Goal commitment** |  |  |  |  |  |
| 114 | Donovan & Radosevich (1998) | Goal commitment | 21 | 2,360 | 21 | 0.36 |
| 115 | Klein, Wesson, Hollenbeck, & Alge (1999) | Goal commitment | 74 |  | 83 | 0.47 |
|  | **Goal intentions** |  |  |  |  |  |
| 116 | Gollwitzer & Sheeran (2007) | Goal intentions on achievement | 63 | 8,461 | 94 | 0.72 |
| 117 | Fuchs & Fuchs (1986) | Long vs short term goals | 18 |  | 96 | 0.64 |
|  |  |  |  |  |  |  |
|  | **Surface Acquiring** |  |  |  |  |  |
|  | **Outlining & transforming** |  |  |  |  |  |
| 118 | Lavery (2008) | Outlining & transforming | 89 |  | 89 | 0.85 |
|  | **Organising** |  |  |  |  |  |
| 119 | Donker et al. (2013) | Organisation | 32 |  | 32 | 0.81 |
| 120 | Dignath, Buettner, & Langfeldt (2008) | Organisation | 50 |  | 50 | 0.75 |
| 121 | Purdie & Hattie (1999) | Organisation | 22 |  | 22 | 0.23 |
|  | **Record keeping** |  |  |  |  |  |
| 122 | Lavery (2008) | Record keeping | 46 |  | 46 | 0.59 |
|  |  | **Influence** | **No. studies** | **No. people** | **No. effects** | **ES** |
| 123 | Dunlosky, Rawson, Marsh, Nathan, & Willingham (2013)) | Reviewing records | 131 |  | 131 | 0.49 |
|  | **Summarisation** |  |  |  |  |  |
| 124 | Dunlosky, Rawson, Marsh, Nathan, & Willingham (2013) | Summarisation | 20 | 1,914 | 157 | 0.57 |
| 125 | Dignath, Buettner, & Langfeldt (2008) | Summarisation | 50 |  | 50 | 0.75 |
|  | **Underlining & highlighting** |  |  |  |  |  |
| 126 | Dunlosky, Rawson, Marsh, Nathan, & Willingham (2013) | Underlining | 16 | 2,070 | 44 | 0.50 |
|  | **Note taking** |  |  |  |  |  |
| 127 | Dunlosky, Rawson, Marsh, Nathan, & Willingham (2013) | Note taking | 5 | 447 | 49 | 0.45 |
| 128 | Purdie & Hattie (1999) | Note taking | 40 |  | 40 | 0.44 |
| 129 | Henk & Stahl (1985) | Note taking | 21 |  | 25 | 0.34 |
| 130 | Kobayashi (2005) | Note taking | 57 |  | 131 | 0.22 |
| 131 | Larwin & Larwin (2013) | Guided notes | 12 | 1,529 | 27 | 0.55 |
| 132 | Larwin, Gorman, & Larwin (2013) | Testing aids (notes, crib sheets, text books) | 15 | 3,146 | 15 | 0.99 |
| 133 | Marzano (1994) | Note taking | 36 |  |  |  |
|  | **Mnemonics** |  |  |  |  |  |
| 134 | Dunlosky, Rawson, Marsh, Nathan, & Willingham (2013) | Mnemonics | 21 | 1,007 | 87 | 0.34 |
| 135 | Runyan (1987) | Mnemonics | 32 | 3,698 | 51 | 0.64 |
| 136 | Kim et al. (2008) | Mnemonics | 8 |  | 14 | 0.45 |
| 137 | Mastropieri & Scruggs (1989) | Mnemonics | 19 |  | 19 | 1.62 |
|  | **Strategy to integrate with prior knowledge** |  |  |  |  |  |
| 138 | Kim et al. (2008) | Strategy to integrate with prior knowledge | 10 |  | 12 | 0.93 |
|  | **Imagery** |  |  |  |  |  |
| 139 | Lavery (2008) | Imagery | 12 | 991 | 59 | 0.45 |
|  | **Working memory training** |  |  |  |  |  |
| 140 | Melby-Lervag & Hulme (2013) | Working memory training | 23 |  | 30 | 0.35 |
| 141 | Linck, Osthus, Koeth, & Bunting (2014) | Working memory training | 79 | 3,707 | 748 | 0.41 |
| 142 | Daneman & Merikle (1996) | Working memory training | 77 | 6,179 | 150 | 0.82 |
|  |  |  |  |  |  |  |
|  | **Surface Consolidation** |  |  |  |  |  |
|  | **Reviewing records** |  |  |  |  |  |
|  |  | **Influence** | **No. studies** | **No. people** | **No. effects** | **ES** |
| 143 | Dunlosky, Rawson, Marsh, Nathan, & Willingham (2013) | Re-reading | 8 | 523 | 84 | 0.49 |
|  | **Practice testing** |  |  |  |  |  |
| 144 | Kulik, Kulik, & Bangert (1984) | Practice testing | 19 |  | 19 | 0.42 |
| 145 | Fuchs & Fuchs (1986) | Examiner familiarity effects | 22 | 1,489 | 34 | 0.28 |
| 146 | Bangert-Drowns, Kulik, & Kulik (1991) | Frequent testing | 35 |  | 35 | 0.23 |
| 147 | Gocmen (2003) | Frequent testing | 78 |  | 233 | 0.40 |
| 148 | Hausknecht, Halpert, Di Paolo, & Moriarty-Gerrard (2007) | Practice and retesting effects | 107 | 134,436 | 107 | 0.26 |
| 149 | Haynie (2007) | Testing on retention learning | 8 |  | 27 | 0.66 |
| 150 | Basol & Johanson (2009) | Frequency of testing | 78 |  | 118 | 0.46 |
| 151 | Phelps (2012) | Effects of testing | 177 | 7,000,000 | 640 | 0.55 |
| 152 | Rowland (2014) | Effects of testing | 61 |  | 159 | 0.50 |
| 153 | Adesope (2013) | Effects of pre-testing | 89 | 11,700 | 226 | 0.63 |
|  | **Spaced vs mass practice** |  |  |  |  |  |
| 154 | Lee & Genovese (1988) | Spaced vs massed practice | 52 |  | 52 | 0.96 |
| 155 | Donovan & Radosevich (1999) | Spaced vs massed practice | 63 |  | 112 | 0.46 |
| 156 | Janiszewski, Noel, & Sawyer (2003) | Spaced vs massed practice | 61 |  | 484 | 0.72 |
| 157 | Cepeda, Pashler, Vul, Wixted, & Rohrer (2009) | Spaced vs massed practice | 184 | 14,811 | 317 | 0.27 |
|  | **Rehearsal and memorisation** |  |  |  |  |  |
| 158 | Lavery (2008) | Rehearsal and memorisation | 99 |  | 99 | 0.57 |
| 159 | Donker et al. (2013) | Rehearsal (Playing flashcards to learn new word) | 10 |  | 10 | 1.39 |
| 160 | Purdie & Hattie (1999) | Memorisation | 23 |  | 23 | 0.23 |
| 161 | Rolhelser-Bennett (1987) | Working memory training | 12 | 1,968 | 78 | 1.28 |
| 162 | Melby-Lervag & Hulme (2013) | Working memory training | 23 |  | 30 | 0.35 |
| 163 | Linck, Osthus, Koeth, & Bunting (2014) | Working memory training | 79 | 3,707 | 748 | 0.41 |
| 164 | Daneman & Merikle (1996) | Working memory training | 77 | 6,179 | 150 | 0.82 |
| 165 | Bos, Ven, Krosebergen, & Luit (2013) | Working memory on math achievement | 68 |  | 288 | 0.62 |
| 166 | Carretti, Borella, Cornoldi, & de Beni (2009) | Working memory on reading achievement | 19 | 1,613 | 19 | 0.71 |
|  | **Teaching test taking & coaching** |  |  |  |  |  |
| 167 | Messick & Jungeblut (1981) | Coaching for SAT | 12 |  | 12 | 0.15 |
|  |  | **Influence** | **No. studies** | **No. people** | **No. effects** | **ES** |
| 168 | Bangert-Drowns, Kulik, & Kulik (1983) | Training in test taking skills | 30 |  | 30 | 0.25 |
| 169 | DerSimonian & Laird (1983) | Coaching on the SAT-M/V | 36 | 15,772 | 36 | 0.07 |
| 170 | Samson (1985) | Training in test taking skills | 24 |  | 24 | 0.33 |
| 171 | Scruggs, White, & Bennion (1986) | Training in test taking skills | 24 |  | 65 | 0.21 |
| 172 | Kalaian & Becker (1986) | Coaching for SAT | 34 |  | 34 | 0.34 |
| 173 | Powers (1986) | Coaching for college admission | 10 |  | 44 | 0.21 |
| 174 | Becker (1990) | Coaching for SAT | 48 |  | 70 | 0.30 |
| 175 | Witt (1993) | Training in test taking skills | 35 |  | 35 | 0.22 |
| 176 | Kulik, Bangert-Drowns, & Kulik (1994) | Coaching for SAT | 14 |  | 14 | 0.15 |
| 177 | Haynie (2007) | Test taking on retention learning | 8 |  | 8 | 0.76 |
|  | **Interleaved practice** |  |  |  |  |  |
| 178 | Dunlosky, Rawson, Marsh, Nathan, & Willingham (2013) | Interleaved practice | 12 | 989 | 65 | 0.21 |
|  | **Effort** |  |  |  |  |  |
| 179 | Donker et al. (2013) | Effort | 15 |  | 15 | 0.77 |
|  | **Time on task** |  |  |  |  |  |
| 180 | Purdie & Hattie (1999) | Time on Task | 36 |  | 36 | 0.24 |
| 181 | Bloom (1976) | Time on task | 11 |  | 28 | 0.75 |
| 182 | Fredrick (1980) | Time on task | 35 |  | 35 | 0.34 |
| 183 | Marzano (2000) | Time on task | 15 |  | 15 | 0.39 |
| 184 | Catts (1992) | Time on task | 18 |  | 37 | 0.19 |
| 185 | Shulruf, Keuskamp, & Timperley (2006) | Taking more coursework | 36 |  | 36 | 0.24 |
| 186 | Cook, Levinson, & Garside (2010) | Time on task | 13 |  | 14 | 1.25 |
| 187 | Crede, Roch, & Kieszczynka (2010) | Class attendance | 90 | 28,034 | 99 | 0.95 |
|  | **Deliberate practice** |  |  |  |  |  |
| 188 | Macnamara, Hambrick, & Oswald (2014) | Deliberate practice | 88 | 11,135 | 88 | 0.43 |
| 189 | Platz, Kopiez, Lehmann, & Wolf (2014) | Deliberate practice | 13 | 788 | 157 | 0.35 |
| 190 | Feltz & Landers (1983) | Mental practice on motor skill learning | 60 | 1,766 | 13 | 1.54 |
|  | **Giving/receiving feedback** |  |  |  | 146 | 0.48 |
| 191 | Lysakowski & Walberg (1980) | Classroom reinforcement | 39 | 4,842 | 102 | 1.17 |
| 192 | Wilkinson (1981) | Teacher praise | 14 |  | 14 | 0.12 |
|  |  | **Influence** | **No. studies** | **No. people** | **No. effects** | **ES** |
| 193 | Walberg (1982) | Cues, and reinforcement | 19 |  | 19 | 0.81 |
| 194 | Lysakowski & Walberg (1982) | Cues, participation and corrective feedback | 54 | 15,689 | 94 | 0.97 |
| 195 | Yeany & Miller (1983) | Diagnostic feedback in college science | 49 |  | 49 | 0.52 |
| 196 | Schmmel (1983) | Feedback from computer instruction | 15 |  | 15 | 0.47 |
| 197 | Getsie, Langer, & Glass (1985) | Rewards and punishment | 89 |  | 89 | 0.14 |
| 198 | Skiba, Casey, & Center (1985) | Nonaversive procedures | 35 |  | 315 | 0.68 |
| 199 | Menges & Brinko (1986) | Student evaluation as feedback | 27 |  | 31 | 0.44 |
| 200 | Rummel & Feinberg (1988) | Extrinsic feedback rewards | 45 |  | 45 | 0.60 |
| 201 | Kulik & Kulik (1988) | Timing of feedback | 53 |  | 53 | 0.33 |
| 202 | Tenenbaum & Goldring (1989) | Cues, and reinforcement | 15 | 522 | 15 | 0.72 |
| 203 | L'Hommedieu, Menges, & Brinko (1990) | Feedback from college student ratings | 28 | 1,698 | 28 | 0.34 |
| 204 | Bangert-Drowns, Kulik, Kulik, & Morgan (1991) | Feedback from tests | 40 |  | 58 | 0.26 |
| 205 | Wiersma (1992) | Intrinsic vs extrinsic rewards | 20 | 865 | 17 | 0.50 |
| 206 | Travlos & Pratt (1995) | Knowledge of results | 17 |  | 17 | 0.71 |
| 207 | Azevedo & Bernard (1995) | Computer presented feedback | 22 |  | 22 | 0.80 |
| 208 | Standley (1996) | Music as reinforcement | 98 |  | 208 | 2.87 |
| 209 | Kluger & DeNisi (1996) | Feedback | 470 | 12,652 | 470 | 0.38 |
| 210 | Neubert (1998) | Goals plus feedback | 16 | 744 | 16 | 0.63 |
| 211 | Swanson & Lussier (2001) | Dynamic assessment (feedback) | 30 | 5,104 | 170 | 1.12 |
| 212 | Miller (2003) | Corrective feedback on learning | 8 |  | 8 | 1.08 |
| 213 | Baker & Dwyer (2005) | Field independent vs field dependent | 11 | 1,341 | 122 | 0.93 |
| 214 | Witt, Wheeless, & Aooen (2006) | Immediacy of teacher feedback | 81 | 24,474 | 81 | 1.15 |
| 215 | Dragon (2009) | Field independent vs field dependent | 35 | 3,082 | 35 | 0.43 |
| 216 | Kleij, Feskens, & Eggen (2015) | Feedback in CAI | 40 | 4,266 | 28 | 0.74 |
| 217 | Lyster & Saito (2010) | Oral feedback on learning | 15 |  | 70 | 0.42 |
| 218 | Li (2010) | Corrective feedback on learning | 28 |  | 28 | 0.61 |
|  | **Help seeking** |  |  |  |  |  |
| 219 | Lavery (2008) | Help seeking | 62 |  | 62 | 0.60 |
|  |  |  |  |  |  |  |
|  | **Deep Acquiring** |  |  |  |  |  |
|  |  | **Influence** | **No. studies** | **No. people** | **No. effects** | **ES** |
|  | **Meta-cognitive strategies** |  |  |  |  |  |
| 220 | Haller, Child, & Walberg (1988) | Metacognitive training programs in reading | 20 | 1,553 | 20 | 0.71 |
| 221 | Chiu (1998) | Metacognitive interventions in reading | 43 | 3,475 | 123 | 0.67 |
| 222 | Donker, de Boer, Dignath, Kostons, & Werf (2013) | Learning strategies on achievement | 58 |  | 180 | 0.66 |
| 223 | Jacob & Parkinson (2015) | Executive functioning | 67 | 15,879 | 291 | 0.36 |
| 224 | Kyriakides, Christoforou, & Charalambous (2013) | Learning strategies on achievement | 167 | 1,182,117 | 167 | 0.63 |
|  | **Elaboration & organisation** |  |  |  |  |  |
| 225 | Donker et al. (2013) | Elaboration | 50 |  | 50 | 0.75 |
|  | **Concept mapping** |  |  |  |  |  |
| 226 | Marzano (1994) | Idea representation | 708 |  | 708 | 0.69 |
| 227 | Moore & Readence (1984) | Graphics organisers in mathematics | 161 |  | 161 | 0.22 |
| 228 | Vazquez & Carballo (1993) | Concept mapping in science | 17 |  | 19 | 0.57 |
| 229 | Horton, McConney, Gallo, Woods, Senn, & Hamelin (1993) | Concept mapping in science | 19 | 1,805 | 19 | 0.45 |
| 230 | Kang (2002) | Graphics organisers in reading with learning disabled | 14 |  | 14 | 0.79 |
| 231 | Kim, Vaughn, Wanzek, & Wei (2004) | Graphics organisers in reading | 21 | 848 | 52 | 0.81 |
| 232 | Nesbit & Adesope (2006) | Concept and knowledge maps | 55 | 5,818 | 67 | 0.55 |
| 233 | Campbell (2009) | Concept making in all subjects | 38 |  | 46 | 0.79 |
| 234 | Dexter & Hughes (2011) | Graphic organisers with learning disabled | 16 | 808 | 55 | 0.91 |
|  | **Elaborative-interrogation** |  |  |  |  |  |
| 235 | Dunlosky, Rawson, Marsh, Nathan, & Willingham (2013) | Elaborative-interrogation | 24 | 2,150 | 164 | 0.42 |
|  | **Strategy monitoring** |  |  |  |  |  |
| 236 | Donker et al. (2013) | Monitoring & control | 81 |  | 81 | 0.71 |
|  | **Self-regulation** |  |  |  |  |  |
| 237 | Ragosta (2010) | Self-regulation with college students | 55 | 6,669 | 93 | 0.71 |
| 238 | Benz (2010) | Self-regulated interventions | 44 | 4,047 | 44 | 0.45 |
| 239 | Lavery (2008) | Self-regulated learning | 30 | 1,937 | 223 | 0.69 |
| 240 | Dignath, Buettner, & Langfeldt (2008) | Self-regulation strategies | 30 | 2,364 | 263 | 0.66 |
| 241 | Benz & Schmitz (2009) | Self-regulated learning | 28 | 4,047 | 28 | 0.37 |
|  |  | **Influence** | **No. studies** | **No. people** | **No. effects** | **ES** |
| 242 | Sitzmann & Ely (2011) | Self-regulation strategies | 369 | 90,380 | 855 | 0.26 |
|  |  |  |  |  |  |  |
|  | **Deep Consolidating** |  |  |  |  |  |
|  | **Evaluation and reflection** |  |  |  |  |  |
| 243 | Donker et al. (2013) | Evaluation & reflection | 54 |  | 54 | 0.75 |
|  | **Via becoming a teacher (peer tutoring)** |  |  |  |  |  |
| 244 | Hartley (1977) | Effects on tutees in math | 29 |  | 50 | 0.63 |
| 245 | Hartley (1977) | Effects on tutors in math | 29 |  | 18 | 0.58 |
| 246 | Cohen, Kulik, & Kulik (1982) | Effects on tutees | 65 |  | 52 | 0.40 |
| 247 | Cohen, Kulik, & Kulik (1982) | Effects on tutors | 65 |  | 33 | 0.33 |
| 248 | Phillips (1983) | Tutorial training of conservation | 302 |  | 302 | 0.98 |
| 249 | Cook, Scruggs, Mastropieri, & Casto (1995) | Handicapped as tutors | 19 |  | 49 | 0.53 |
| 250 | Cook, Scruggs, Mastropieri, & Casto (1995) | Handicapped as tutees | 19 |  | 25 | 0.58 |
| 251 | Mathes & Fuchs (1991) | Peer tutoring in reading | 11 |  | 74 | 0.36 |
| 252 | Batya, Vaughn, Hughes, & Moody (2000) | Peer tutoring in reading | 32 | 1,248 | 216 | 0.41 |
| 253 | Elbaum, Vaughn, Hughes, & Moody (2000) | One-one tutoring programs in reading | 29 | 325 | 216 | 0.67 |
| 254 | Rohrbeck, Ginsburg-Block, Fantuzzo, & Miller (2003) | Peer assisted learning | 90 |  | 90 | 0.59 |
| 255 | Erion (2006) | Parent tutoring children | 32 |  | 32 | 0.82 |
| 256 | Ginsburg-Block, Rohrbeck, & Fantuzzo (2006) | Peer-assisted learning | 28 |  | 26 | 0.35 |
| 257 | Leung (2014) | Peer tutoring | 72 | 15,517 | 72 | 0.39 |
| 258 | Kunsch, Jitendra, & Sood (2007) | Peer mediated instruction in math with LD students | 17 | 1,103 | 17 | 0.47 |
|  | **Self-verbalisation & self-questioning** |  |  |  |  |  |
| 259 | Dunlosky, Rawson, Marsh, Nathan, & Willingham (2013) | Self-verbalisation & Self-questioning | 113 | 3,098 | 1,150 | 0.64 |
| 260 | Rock (1985) | Special ed self-instructional training | 47 | 1,398 | 684 | 0.51 |
| 261 | Duzinski (1987) | Self-verbalising instruction training | 45 |  | 377 | 0.84 |
| 262 | Huang (1991) | Student self-questioning | 21 | 1,700 | 89 | 0.58 |
|  | **Self-monitoring** |  |  |  |  |  |
| 263 | Lavery (2008) | Self-monitoring | 154 |  | 154 | 0.45 |
|  | **Self-verbalising the steps in a problem** |  |  |  |  |  |
|  |  | **Influence** | **No. studies** | **No. people** | **No. effects** | **ES** |
| 264 | Lavery (2008) | Self-verbalising the steps in a problem | 124 |  | 124 | 0.62 |
| 265 | Marzano (2000) | Process specification & monitoring | 15 |  | 15 | 0.30 |
| 266 | Marzano (2000) | Dispositional monitoring | 15 |  | 15 | 0.30 |
|  | **Self-consequences** |  |  |  |  |  |
| 267 | Lavery (2008) | Self-consequences | 75 |  | 75 | 0.70 |
|  | **Self-explanation** |  |  |  |  |  |
| 268 | Lavery (2008) | Self-explanation | 8 | 533 | 69 | 0.50 |
|  | **Seeking help from peers** |  |  |  |  |  |
| 269 | Dignath, Buettner, & Langfeldt (2008) | Seeking help from peers | 21 |  | 21 | 0.83 |
|  | **Collaborative/cooperative learning** |  |  |  |  |  |
| 270 | Johnson, Maruyama, Johnson, Nelson, & Skon (1981) | Cooperative learning | 122 |  | 183 | 0.73 |
| 271 | Rolhelser-Bennett (1987) | Cooperative learning | 23 | 4,002 | 78 | 0.48 |
| 272 | Hall (1988) | Cooperative learning | 22 | 10,022 | 52 | 0.31 |
| 273 | Stevens & Slavin (1991) | Cooperative learning | 4 |  | 4 | 0.48 |
| 274 | Spuler (1993) | Cooperative learning in math | 19 | 6,137 | 19 | 0.54 |
| 275 | Othman (1996) | Cooperative learning in math | 39 |  | 39 | 0.27 |
| 276 | Howard (1996) | Scripted cooperative learning | 13 |  | 42 | 0.37 |
| 277 | Suri (1997) | Cooperative learning in math | 27 |  | 27 | 0.63 |
| 278 | Bowen (2000) | Cooperative learning in high school chemistry | 37 | 3,000 | 49 | 0.51 |
| 279 | Neber, Finsterwald, & Urban (2001) | Cooperative learning with gifted | 12 |  | 314 | 0.13 |
| 280 | McMaster & Fuchs (2002) | Cooperative learning | 15 | 864 | 49 | 0.30 |
| 281 | Stoner (2004) | Cooperative learning | 22 | 6,455 | 22 | 0.14 |
| 282 | Romero (2009) | Cooperative learning | 32 |  | 52 | 0.31 |
| 283 | Williams (2009) | Collaborative learning | 29 | 3,029 | 29 | 0.29 |
| 284 | Igel (2010) | Cooperative learning | 20 | 2,412 | 20 | 0.44 |
| 285 | Nunnery, Chappell, & Arnold (2013) | Cooperative learning in math | 15 |  | 15 | 0.16 |
| 286 | Kyndt, Raes, Lismont, Timmers, Cascallar, & Dochy (2013) | Cooperative learning | 43 |  | 51 | 0.54 |
|  | **Critical thinking techniques** |  |  |  |  |  |
| 287 | Abrami, Bernard, Borokhovski, Wade, Surkes, Tamim, & Zhang (2008) | Critical thinking interventions | 117 | 20,698 | 161 | 0.34 |
|  |  | **Influence** | **No. studies** | **No. people** | **No. effects** | **ES** |
|  | **Classroom discussion** |  |  |  |  |  |
| 288 | Murphy, Wilkinson, Soter, & Hennessey (2011) | Fostering classroom discussion | 42 |  | 42 | 0.82 |
|  | **Problem solving teaching** |  |  |  |  |  |
| 289 | Marzano (2000) | Problem solving | 343 |  | 343 | 0.54 |
| 290 | Xin & Jitendra (1999) | Word problem solving in reading | 14 |  | 653 | 0.89 |
| 291 | Swanson (2001) | Programs to enhance problem solving | 58 |  | 58 | 0.82 |
| 292 | Marcucci (1980) | Problem solving in math | 33 |  | 237 | 0.35 |
| 293 | Curbello (1984) | Problem solving on science and math | 68 | 10,629 | 343 | 0.54 |
| 294 | Almeida & Denham (1984) | Interpersonal problem solving | 18 | 2,398 | 18 | 0.72 |
| 295 | Mellinger (1991) | Increasing cognitive flexibility | 25 |  | 35 | 1.13 |
| 296 | Hembree (1992) | Problem solving instructional methods | 55 |  | 55 | 0.33 |
| 297 | Tocanis, Ferguson-Hessler, & Broekkamp (2001) | Problem solving in science | 22 | 2,208 | 31 | 0.59 |
| 298 | Johnson & Johnson (2009) | Conflict based teaching | 39 |  | 39 | 0.80 |
| 299 | Zheng, Flynn & Swanson (2011) | Problem solving with math disabilities | 8 |  | 8 | 0.78 |
|  |  |  |  |  |  |  |
|  | **Transfer** |  |  |  |  |  |
|  | **Far transfer** |  |  |  |  |  |
| 300 | Rayner Bernard, & Osana (2013) | Far transfer in math | 53 |  | 116 | 0.80 |
|  | **Seeing patterns to new situations** |  |  |  |  |  |
| 301 | Marzano (2000) | Experimental inquiry | 6 |  | 6 | 1.14 |
|  | **Similarities and differences** |  |  |  |  |  |
| 302 | Marzano (2000) | Identifying similarities and differences | 51 |  | 51 | 1.32 |

Appendix B. References for the meta-analysis cited in Appendix A.

Abrami, P. C., Bernard, R. M., Borokhovski, E., Wade, A. C., Surkes, M. A.,Tamim, R*.,* & Zhang, D. (2008, May). Instructional interventions affecting critical thinking skills and dispositions: A stage 1 meta-analysis. *Review of Educational Research, 78 (4),* 1102-1134.

Adesope, O. O**.,** Trevisan, D. A., & Trevisan, M. (2013). *A meta-analysis of the testing effect.* Paper presented at the annual American Educational Research Association Annual Meeting, San Francisco, CA.

Almeida, M. C., & Denham, S. A. (1984, April). *Interpersonal cognitive problem-solving: A meta-analysis.* Paper presented at the Annual Meeting of the Eastern Psychological Association, Baltimore.

Asencio, C. E. (1984). *Effects of behavioral objectives on student achievement: A meta-analysis of findings.* Unpublished doctoral dissertation, The Florida State University, FL.

Astill, R.G., Van der Heijden, Van IJzendoom, & Van Someren, E.J.W. (2012). Sleep, cognition, and behavioral problems in school-age children: A century of research meta-analyzed. *Psychological Bulletin, 138 (6*), 1109-1138.

Azevedo, R., & Bernard, R.M. (1995, April). *The effects of computer-presented feedback on learning from computer-based instruction: A meta-analysis*. Paper present at the Annual Meeting of the American Educational Research Association. CA: San Francisco. ERIC document 385 235

Baker, R. M., & Dwyer, F. (2005). Effect of instructional strategies and individual differences: A meta-analytic assessment. *International Journal of Instructional Media, 32*(1), 69.

Bangert, R. L., Kulik, J. A., & Kulik, C. L. C. (1983). Individualized systems of instruction in secondary schools. *Review of Educational Research, 53*(2), 143–158.

Bangert-Drowns, R. L., Kulik, J. A., & Kulik, C. L. C. (1991). Effects of frequent classroom testing. *Journal of Educational Research, 85*(2), 89-99.

Bangert-Drowns, R. L., Kulik, C. L. C., Kulik, J. A., & Morgan, M.T. (1991).The instructional effect of feedback in test-like events. *Review of Educational Research, 61*(2), 213–238.

Başol, G., & Johanson, G. (2009). Effectiveness of frequent testing over achievement: A meta analysis study. International Journal of Human Sciences, 6(2), 99-121.

Abrami, P. C., Bernard, R. M., Borokhovski, E., Wade, A., Surkes, M. A., Tamim, R., & Zhang, D. (2008). Instructional interventions affecting critical thinking skills and dispositions: A stage 1 meta-analysis. *Review of Educational Research, 78(4*), 1102-1134.

Becker, B. J. (1990). Coaching for the scholastic aptitude test: Further synthesis and appraisal. *Review of Educational Research, 60*(3), 373–417.

Benz, B. F. (2010). *Improving the quality of e-learning by enhancing self-regulated learning. A synthesis of research on self-regulated learning and an implementation of a scaffolding concept*. Unpublished doctoral dissertation, TU Darmstadt.

Bloom, B. S. (1976). *Human characteristics and school learning*. New York: McGraw-Hill.

Bloom, B. S. (1984).The search for methods of group instruction as effective as one-to-one tutoring. *Educational Leadership, 41*(8), 4–17.

Boulanger, F. D. (1981). Instruction and science learning: A quantitative synthesis. *Journal of Research in Science Teaching, 18*(4), 311–327.

Bourhis, J., & Allen, M. (1992). Meta-analysis of the relationship between communication apprehension and cognitive performance. *Communication Education, 41*(1), 68–76.

Bowen, C.W. (2000). A quantitative literature review of cooperative learning effects on high school and college chemistry achievement. *Journal of Chemical Education, 77*(1), 116–119.

Bradford, J. W. (1990). *A meta-analysis of selected research on student attitudes towards mathematics.* Unpublished doctoral dissertation, University of Iowa, Iowa City, IA.

Burnette, J. L., O'Boyle, E. H., Van Epps, E. M., Pollack, J. M., & Finkel, E. J. (2013). Mind-sets matter: A meta-analytic review of implicit theories and self-regulation. *Psychological Bulletin*, *139*(3), 655-701.

Burns, M. K. (2004). Empirical analysis of drill ratio research: Refining the instructional level for drill tasks. *Remedial and Special Education, 25*(3), 167–173.

Campbell, L.O. (2009). *A meta-analytical review of Novak’s concept mapping*. Unpublished doctoral dissertation, Regent University.

Carpenter, S.L. (2007). *A comparison of the relationships of students’ self-efficacy, goal orientation, and achievement across grade levels: A meta-analysis*. Unpublished doctoral dissertation, Simon Fraser University, Canada.

Carretti, B., Borella, E., Cornoldi, C., & De Beni, R. (2009). Role of working memory in explaining the performance of individuals with specific reading comprehension difficulties: A meta-analysis. *Learning and Individual Differences*, *19*(2), 246-251.

Catts, R. (1992). *The integration of research findings: A review of meta-analysis methodology and an application to research on the effects of knowledge of objectives*. Unpublished doctoral dissertation, University of Sydney, Sydney, Australia.

Cepeda, N. J., Pashler, H., Vul, E., Wixted, J. T., & Rohrer, D. (2006). Distributed practice in verbal recall tasks: A review and quantitative synthesis. *Psychological Bulletin, 132*(3), 354–380.

Cerasoli, C. P., Nicklin, J. M., & Ford, M. T. (2014). Intrinsic motivation and extrinsic incentives jointly predict performance: A 40-year meta-analysis. *Psychological Bulletin*, *140*(4), 980.

Chang, Y., Labban, J., Gapin, J., & Etnier, J. (2012). The effects of acute exercise on cognitive performance: a meta-analysis. *Brain research, 1453,* 87-101.

Chen, S., Chen, A., & Zhu, X. (2012). Are K–12 Learners Motivated in Physical Education? A Meta-Analysis. *Research quarterly for exercise and sport, 83(1*), 36-48.

Chid ester, T. R., & Grigsby, W.C. (1984). A meta-analysis of the goal setting-performance literature. *Academy of Management Proceedings*, 202–206.

Chiu, C. W. T. (1998, April). *Synthesizing metacognitive interventions: What training characteristics can improve reading performance?* Paper presented at the Annual Meeting of the American Educational Research Association San Diego, CA.

Cohen, P.A., Kulik, J.A., & Kulik, C.L. C. (1982). Educational outcomes of tutoring: A meta-analysis of findings. *American Educational Research Journal, 19*(2), 237–248.

Cook, D. A., Levinson, A. J., & Garside, S. (2010). Time and learning efficiency in Internet-based learning: a systematic review and meta-analysis. *Advances in Health Sciences Education*, *15*(5), 755-770.

Cook, S. B., Scruggs, T. E., Mastropieri, M. A., & Casto, G. C. (1985). Handicapped students as tutors. *The Journal of Special Education, 19(4),* 483-492.

Credé, M., & Phillips, L. A. (2011). A meta-analytic review of the Motivated Strategies for Learning Questionnaire. *Learning and Individual Differences*, *21*(4), 337-346.

Credé, M., Roch, S. G., & Kieszczynka, U. M. (2010). Class attendance in college a meta-analytic review of the relationship of class attendance with grades and student characteristics. *Review of Educational Research*, *80*(2), 272-295.

Crissman, J. K. (2006). *The design and utilization of effective worked examples: A meta-analysis.* Unpublished doctoral dissertation, The University of Nebraska - Lincoln, Nebraska, United States.

Curbelo, J. (1984). *Effects of problem-solving instruction on science and mathematics student achievement: A* *meta-analysis of findings.* Unpublished doctoral dissertation, The Florida State University, FL.

Daneman, M., & Merikle, P. M. (1996). Working memory and language comprehension: A meta- analysis. *Psychonomic Bulletin and Review, 3*(4), 422–433.

DerSimonian, R., & Laird, N. M. (1983). Evaluating the effect of coaching on SAT scores: A meta- analysis. *Harvard Educational Review, 53*(1), 1–15.

Dewald, J. F., Meijer, A. M., Oort, F. J., Kerkhof, G. A., & Bögels, S. M. (2010). The influence of sleep quality, sleep duration and sleepiness on school performance in children and adolescents: a meta-analytic review. *Sleep medicine reviews*, *14*(3), 179-189.

Dexter, D. D., & Hughes, C. A. (2011). Graphic organizers and students with learning disabilities: A meta-analysis. *Learning Disability Quarterly*, *34*(1), 51-72.

Dignath, C., Buettner, G., & Langfeldt, H. P. (2008). How can primary school students learn self-regulated learning strategies most effectively?: A meta-analysis on self-regulation training programmes. *Educational Research Review,* 3(2), 101-129.

Donker, A. S., de Boer, H., Kostons, D., van Ewijk, C. D., & Van der Werf, M. P. C. (2014). Effectiveness of learning strategy instruction on academic performance: A meta-analysis. *Educational Research Review*, *11*, 1-26.

Donovan, J. J., & Radosevich, D. J. (1998). The moderating role of goal commitment on the goal difficulty–performance relationship: A meta-analytic review and critical reanalysis. *Journal of Applied Psychology*, *83*(2), 308.

Donovan, J. J., & Radosevich, D. J. (1999). A meta-analytic review of the distribution of practice effect: Now you see it, now you don’t. *Journal of Applied Psychology, 84*(5), 795–805.

Dragon, K. (2009). *Field dependence and student achievement in technology-based learning: A meta-analysis*. Unpublished doctoral dissertation, University of Alberta, Canada.

Duncan, G. J., Dowsett, C. J., Claessens, A., Magnuson, K., Huston, A. C., Klebanov, P., Pagani, L.S., Feinstein, L., Engel, M., Brooks-Gunn, J., Sexton, H., Duckworth, K., & Japel, C.. (2007). School readiness and later achievement. *Developmental Psychology, 43*(6), 1428–1446.

Dunlosky, J., Rawson, K. A., Marsh, E. J., Nathan, M. J., & Willingham, D. T. (2013). Improving students’ learning with effective learning techniques promising directions from cognitive and educational psychology. *Psychological Science in the Public Interest*, *14*(1), 4-58.

Duzinski, G. A. (1987). *The educational utility of cognitive behavior modification strategies with children: A* *quantitative synthesis.* Unpublished doctoral dissertation, University of Illinois at Chicago, IL.

Elbaum, B., Vaughn, S., Hughes, M.T., & Moody, S.W. (2000). How effective are one-to-one tutoring programs in reading for elementary students at risk for reading failure? A meta-analysis of the intervention research. *Journal of Educational Psychology, 92*(4), 605–619.

Erion, J. (2006). Parent tutoring: A meta-analysis. *Education and Treatment of Children, 29*(1), 79–106.

Ernst, M. L. M. (2001). *Infant cognition and later intelligence.* Unpublished doctoral dissertation, Loyola University of Chicago, IL.

Etnier, J. L., Nowell, P. M., Landers, D. M., & Sibley, B. A. (2006). A meta-regression to examine the relationship between aerobic fitness and cognitive performance. *Brain Research Reviews, 52*(1), 119–130.

Etnier, J. L., Salazar, W., Landers, D. M., Petruzzello, S. J., Han, M., & Nowell, P. (1997).The influence of physical fitness and exercise upon cognitive functioning: A meta-analysis. *Journal of Sport and Exercise Psychology, 19*(3), 249–277.

Fedewa, A. L., & Ahn, S. (2011). The effects of physical activity and physical fitness on children's achievement and cognitive outcomes: a meta-analysis. *Research Quarterly for Exercise and Sport*, *82*(3), 521-535.

Feltz, D. L., & Landers, D. M. (1983). The effects of mental practice on motor skill learning and performance: A meta-analysis. *Journal of Sport Psychology, 5*, 25–57.

Findley, M. J., & Cooper, H. M. (1983). Locus of control and academic achievement: A literature review. *Journal of Personality and Social Psychology, 44*(2), 419–427.

Fitzgerald, S.W. (1996). *The relationship between anxiety and statistics achievement: A meta-analysis.* Unpublished doctoral dissertation, University of Toledo.

Fredrick, W. C. (1980). Instructional time. *Evaluation in Education, 4*, 117–118

Friso-van den Bos, I., van der Ven, S. H., Kroesbergen, E. H., & van Luit, J. E. (2013). Working memory and mathematics in primary school children: A meta-analysis. *Educational research review*, *10*, 29-44.

Fuchs, L. S., & Fuchs, D. (1985). *The effect of measuring student progress toward long vs. short-term goals: A meta-analysis*. ERIC Document TM 850 615.

Fuchs, D., & Fuchs, L. S. (1986).Test procedure bias: A meta-analysis of examiner familiarity effects. *Review of Educational Research, 56*(2), 243–262.

Galland, B., Spruyt, K., Dawes, P., McDowall, P. S., Elder, D., & Schaughency, E. (2015). Sleep Disordered Breathing and Academic Performance: A Meta-analysis. *Pediatrics*, *136*(4), e934-e946.

Getsie, R. L., Langer, P., & Glass, G.V. (1985). Meta-analysis of the effects of type and combination of feedback on children’s discrimination learning. *Review of Educational Research, 55*(1), 9–22.

Ginsburg-Block, M. D., Rohrbeck, C. A., & Fantuzzo, J.W. (2006). A meta-analytic review of social, self-concept, and behavioral outcomes of peer-assisted learning. *Journal of Educational Psychology,* *98*(4), 732–749.

Gocmen, G. B. (2003). *Effectiveness of frequent testing over academic achievement: A meta-analysis study.* Unpublished doctoral dissertation, Ohio University, Ohio, United States.

Gollwitzer, P. M., & Sheeran, P. (2006). Implementation intentions and goal achievement: A meta- analysis of effects and processes. *Advances in Experimental Social Psychology, 38*, 69–119.

Hall, L. E. (1988). *The effects of cooperative learning on achievement: A meta-analysis.* Unpublished Ed.D., University of Georgia, GA.

Haller, E. P., Child, D. A., & Walberg, H. J. (1988). Can comprehension be taught? A quantitative synthesis of “metacognitive” studies. *Educational Researcher, 17*(9), 5–8.

Harris, M.M. (1987). *Meta-analyses of test anxiety among college student*. Unpublished doctoral dissertation, University of Ohio.

Hartley, S. S. (1977). *Meta-analysis of the effects of individually paced instruction in mathematics.* Unpublished doctoral dissertation, University of Colorado at Boulder, CO.

Hattie, J.A.C., & Clinton, J. (2012). Physical activity is not related to performance at school. *Archives of Pediatrics & Adolescent Medicine*,*166*(7), 678-679.

Hattie, J.A.C., & Hansford, B.C. (1982, November). *Personality and achievement: What relationship with achievement*. Paper presented at the Australian Association for Research in Education. Brisbane.

Hausknecht, J. P., Halpert, J. A., Di Paolo, N.T., & Gerrard, M. O. M. (2007). Retesting in selection: A meta-analysis of coaching and practice effects for tests of cognitive ability. *Journal of Applied Psychology, 92*(2), 373–385.

Haynie, W. J. (2007). Effects of test taking on retention learning in technology education: A meta- analysis. *Journal of Technology Education, 18*(2), 24–36.

Hembree, R. (1988). Correlates, causes, effects, and treatment of test anxiety. *Review of Educational* *Research, 58*(1), 47–77.

Hembree, R. (1992). Experiments and relational studies in problem solving: A meta-analysis. *Journal for Research in Mathematics Education, 23*(3), 242–273.

Henk, W. A., & Stahl, N. A. (1985, November). *A meta-analysis of the effect of notetaking on learning from lecture. College reading and learning assistance* Paper presented at the Annual Meeting of the National Reading Conference, St. Petersburg Beach, FL.

Holden, G.W., Moncher, M. S., Schinke, S. P., & Barker, K. M. (1990). Self-efficacy of children and adolescents: A meta-analysis. *Psychological Reports, 66*(3, Pt 1), 1044–1046.

Horton, P. B., McConney, A. A., Gallo, M., Woods, A. L., Senn, G. J., & Hamelin, D. (1993). An investigation of the effectiveness of concept mapping as an instructional tool. *Science Education*, *77*(1), 95-111.

Howard, B. C. (1996, February). *A meta-analysis of scripted cooperative learning.* Paper presented at the Annual Meeting of the Eastern Educational Research Association, Boston, MA.

Huang, Z. (1991). *A meta-analysis of student self-questioning strategies.* Unpublished doctoral dissertation, Hofstra University, NY.

Huang, C. (2011). Self-concept and academic achievement: A meta-analysis of longitudinal relations. *Journal of School Psychology*, *49*(5), 505-528.

Hulleman, C. S., Schrager, S. M., Bodmann, S. M., & Harackiewicz, J. M. (2010). A meta-analytic review of achievement goal measures: Different labels for the same constructs or different constructs with similar labels? *Psychological Bulletin*, *136*(3), 422.

Igel, C. C. (2010). The effect of cooperative learning instruction on K-12 student learning: A meta-analysis of quantitative studies from 1998-2009. University of Virginia.

Jacob, R., & Parkinson, J. (2015). The potential for school based interventions that target executive function to improve academic achievement: A review. *Review of Educational Research, 85*, 1-41.

Janiszewski, C., Noel, H., & Sawyer, A. G. (2003). A meta-analysis of the spacing effect in verbal learning: Implications for research on advertising repetition and consumer memory. *Journal of consumer research*, *30*(1), 138-149.

Johnson, D.W., & Johnson, R.T. (2009). Energizing learning: The instructional power of conflict. *Educational Researcher 38 (10*), 37-51.

Johnson, D. W., Maruyama, G., Johnson, R. T., Nelson, D., & Skon, L. (1981). Effects of cooperative, competitive, and individualistic goal structures on achievement: A meta-analysis. *Psychological Bulletin, 89*(1), 47–62.

Kalaian, S., & Becker, B. J. (1986, April). *Effects of coaching on Scholastic Aptitude Test (SAT) performance: A multivariate meta-analysis approach.* Paper presented at the Annual Meeting of the American Educational Research Association, San Francisco, CA.

Kalechstein, A. D., & Nowicki, S., Jr. (1997). A meta-analytic examination of the relationship between control expectancies and academic achievement: an 11-year follow-up to Findley and Cooper. *Genetic, Social, and General Psychology Monographs, 123*(1), 27–56.

Kämpfe, J., Sedlmeier, P., & Renkewitz, F. (2010). The impact of background music on adult listeners: A meta-analysis. *Psychology of Music*, *39*, 424-448.

Kang, O.-R. (2002). *A meta-analysis of graphic organizer interventions for students with learning disabilities.* Unpublished doctoral dissertation University of Oregon, OR.

Karich, A.C., Bruns, M.K., & Maki, K.E. (2014). Updated meta-analysis of learner control within educational technology. *Review of Educational Research, 84 (3),* 392-410.

Kavale, K. A., & Nye, C. (1985). Parameters of learning disabilities in achievement, linguistic, neuropsychological, and social/behavioral domains. *Journal of Special Education, 19*(4), 443–458.

Kim, A. H., Vaughn, S., Wanzek, J., & Wei, S. (2004). Graphic organizers and their effects on the reading comprehension of students with LD:A synthesis of research. *Journal of Learning Disabilities,* *37*(2), 105–118.

Kim, D., Kim, C., Lee, K., Park, J., Hong, S., & Kim, H. (2008).Effects of cognitive learning strategies for Korean Learners: A meta-analysis. *Asia Pacific Education Review, 9(4),* 409-422.

Klauer, K. J. (1981). Zielorientiertes lehren und lernen bei lehrtexten. Eine metaanalyse [Goal oriented teaching and learning in scholarly texts. A Meta-analysis]. *Unterrichtswissenschaft, 9*, 300–318.

van der Kleij, F.M., Feskens, R.C.W., & Eggen, T.J.H.M. (2015). Effects of feedback in a computer-based learning environment on students’ learning outcomes: A meta-analysis.  *Review of Educational Research, 85(4*), 1-37.

Klein, H. J., Wesson, M. J., Hollenbeck, J. R., & Alge, B. J. (1999). Goal commitment and the goal- setting process: Conceptual clarification and empirical synthesis. *Journal of Applied Psychology,* *84*(6), 885–896.

Kluger, A. N., & DeNisi, A. (1996).The effects of feedback interventions on performance: A historical review, a meta-analysis, and a preliminary feedback intervention theory. *Psychological Bulletin,* *119*(2), 254.

Kobayashi, K. (2006). Combined effects of note-taking: Reviewing on learning and the enhancement through Interventions: A meta-analytic review. *Educational Psychology, 26*(3), 459–477.

Kozlow, M. J., & White, A. L. (1980). Advance organiser research. *Evaluation in Education, 4*, 47–48.

Kulik, J. A., & Kulik, C. L. C. (1988). Timing of feedback and verbal learning. *Review of Educational* *Research, 58*(1), 79–97.

Kulik, J. A., Bangert-Drowns, R. L., & Kulik, C.-L. C. (1984). Effectiveness of coaching for aptitude tests. *Psychological Bulletin, 95*(2), 179-188.

Kulik, J. A., Kulik, C. L. C., & Bangert, R. L. (1984). Effects of practice on aptitude and achievement test scores. *American Educational Research Journal, 21*(2), 435–447.

Kuncel, N. R., Hezlett, S. A., & Ones, D. S. (2001). A comprehensive meta-analysis of the predictive validity of the graduate record examinations: Implications for graduate student selection and performance. *Psychological Bulletin, 127*(1), 162–181.

Kunsch, C. A., Jitendra, A. K., & Sood, S. (2007). The effects of peer-mediated instruction in mathematics for students with learning problems: A research synthesis. *Learning Disabilities Research and Practice, 22*(1), 1–12.

Kyndt, E., Raes, E., Lismont, B., Timmers, F., Cascallar, E., & Dochy, F. (2013). A meta-analysis of the effects of face-to-face cooperative learning. Do recent studies falsify or verify earlier findings? *Educational Research Review*, *10*, 133-149.

Kyriakides, L., Christoforou, C., & Charalambous, C. Y. (2013). What matters for student learning outcomes: A meta-analysis of studies exploring factors of effective teaching. *Teaching and Teacher Education*, *36*, 143-152.

Larwin, K., & Larwin, D. (2013). The Impact of Guided Notes on Post-Secondary Student Achievement: A Meta-Analysis. *International Journal of Teaching and Learning in Higher Education*, *25*(1), 47-58.

Larwin, K. H., Gorman, J., & Larwin, D. A. (2013). Assessing the Impact of Testing Aids on Post-Secondary Student Performance: A Meta-Analytic Investigation. *Educational Psychology Review*, *25*(3), 429-443.

Lavery, L. (2008). *Self-regulated learning for academic success: An evaluation of instructional techniques.* Unpublished doctoral dissertation, The University of Auckland, Auckland.

Lee,T. D., & Genovese, E. D. (1988). Distribution of practice in motor skill acquisition: Learning and performance effects reconsidered. *Research Quarterly for Exercise and Sport, 59*(4), 277–287.

Leung, K.C. (2014). Preliminary empirical model of crucial determinants of best practice for peer tutoring on academic achievement. Journal of Educational Psychology, 107(2), 558-579.

L’Hommedieu, R., Menges, R. J., & Brinko, K. T. (1990). Methodological explanations for the modest effects of feedback from student ratings. *Journal of Educational Psychology, 82*(2), 232–241.

Li, S. (2010). The Effectiveness of Corrective Feedback in SLA: A Meta‐Analysis. *Language Learning*, *60*(2), 309-365.

Linck, J. A., Osthus, P., Koeth, J. T., & Bunting, M. F. (2014). Working memory and second language comprehension and production: A meta-analysis. *Psychonomic Bulletin & Review*, *21*(4), 861-883.

Lott, G.W. (1983).The effect of inquiry teaching and advance organizers upon student outcomes in science education. *Journal of Research in Science Teaching, 20*(5), 437–451.

Luiten, J., Ames, W., & Ackerman, G. (1980). A meta-analysis of the effects of advance organizers on learning and retention. *American Educational Research Journal, 17*(2), 211–218.

Lysakowski, R. S., & Walberg, H. J. (1980). Classroom reinforcement. *Evaluation in Education, 4*, 115–116.

Lysakowski, R. S., & Walberg, H. J. (1982). Instructional effects of cues, participation, and corrective feedback: A quantitative synthesis. *American Educational Research Journal, 19*(4), 559–578.

Lyster, R., & Saito, K. (2010). Oral feedback in classroom SLA. *Studies in Second Language Acquisition*, *32*(02), 265-302.

Ma, X. (1999). A meta-analysis of the relationship between anxiety toward mathematics and achievement in mathematics. *Journal for Research in Mathematics Education, 30*(5), 520–541.

Ma, X., & Kishor, N. (1997).Assessing the relationship between attitude toward mathematics and achievement in mathematics: A meta-analysis. *Journal for Research in Mathematics Education, 28*(1), 26–47.

Macnamara, B. N., Hambrick, D. Z., & Oswald, F. L. (2014). Deliberate practice and performance in music, games, sports, education, and professions a meta-analysis. *Psychological Science*, *25*(8), 1608-1618.

Mahar, C. L. (1992). *Thirty years after Ausubel: An updated meta-analysis of advance organizer research.* Unpublished doctoral dissertation, University of Illinois at Urbana, Champaign, IL.

Marcucci, R. G. (1980). *A meta-analysis of research on methods of teaching mathematical problem solving.* Unpublished doctoral dissertation, The University of Iowa, IA.

Marzano, R. J. (1998). *A theory-based meta-analysis of research on instruction*. Aurora, CO: Mid-Continent

Marzano, R. J., Gaddy, B. B., & Dean, C. (2000). *What works in classroom instruction*. Aurora, CO: Mid-Continent Research for Education and Learning.

Marzano, R. J., Pickering, D. J., & Pollock, J. E. (2001). *Classroom instruction that works* (Vol. 5). Alexandria, VA: Association for Supervision and Curriculum Development.

Mastropieri, M., & Scruggs, T. (1989). Constructing more meaningful relationships: Mnemonic instruction for special populations. *Educational Psychology Review, 1*(2), 83–111.

Mathes, P. G., & Fuchs, L. S. (1994).The efficacy of peer tutoring in reading for students with mild disabilities: A best-evidence. *School Psychology Review, 23*(1), 59.

McMaster, K. N., & Fuchs, D. (2002). Effects of cooperative learning on the academic achievement of students with learning disabilities: An update of Tateyama-Sniezek’s review. *Learning Disabilities Research and Practice, 17*(2), 107–117.

Melby-Lervåg, M., & Hulme, C. (2013). Is working memory training effective? A meta-analytic review. *Developmental psychology*, *49*(2), 270.

Mellinger, S. F. (1991). *The development of cognitive flexibility in problem-solving: Theory and application.* Unpublished doctoral dissertation, The University of Alabama, AL.

Menges, R. J., & Brinko, K. T. (1986, April). *Effects of student evaluation feedback: A meta-analysis of higher education research.* Paper presented at the Annual Meeting of the American Educational Research Association, San Francisco, CA.

Mentor, A. J., Steel, R. P., & Karen, R. J. (1987).A meta-analytic study of the effects of goal setting on task performance: 1966–1984. *Organizational Behavior and Human Decision Processes, 39*(1), 52–83.

Mesick, S., & Jungeblut, A. (1981).Time and method in coaching for the SAT. *Psychological Bulletin,* *89*(2), 191–216.

Miller, P. C. (2003). *The effectiveness of corrective feedback: A meta-analysis*. Unpublished doctoral dissertation, Purdue University.

Miloslavic, S.A. (2009). *Antecedents and consequences of goal commitment: A meta-analysis.* Unpublished doctoral dissertation, Florida Institute of Technology, Florida.

Moon, C. E., Render, G. F., & Pendley, D.W. (1985, March-April). *Relaxation and educational outcomes: A meta-analysis.* Paper presented at the Annual Meeting of the American Educational Research Association, Chicago, IL.

Moore, D. W., & Readence, J. E. (1984). A quantitative and qualitative review of graphic organizer research. *Journal of Educational Research, 78*(1), 11–17.

Muller, J. C., Gullung, P., & Bocci, P. (1988). Concept de soi et performance scolaire: Une meta-analyse [Self-concept and academic performance: A meta-analysis]. *Orientation Scolaire et Professionnelle, 17*, 53–69.

Multon, K. D., Brown, S. D., & Lent, R. W. (1991). Relation of self-efficacy beliefs to academic outcomes: A meta-analytic investigation. *Journal of Counseling Psychology, 38*(1), 30–38.

Murphy, P. K., Wilkinson, I. A., Soter, A. O., Hennessey, M. N., & Alexander, J. F. (2009). Examining the effects of classroom discussion on students’ comprehension of text: A meta-analysis. *Journal of Educational Psychology, 101(3),* 740.

Neber, H., Finsterwald, M., & Urban, N. (2001). Cooperative learning with gifted and high-achieving students: A review and meta-analyses of 12 studies. *High Ability Studies*, *12*(2), 199-214.

Nesbit, J. C., & Adesope, O. O. (2006). Learning with concept and knowledge maps: A meta-analysis. *Review of Educational Research, 76*(3), 413–448.

Neubert, M. J. (1998). The value of feedback and goal setting over goal setting alone and potential moderators of this effect: A meta-analysis. *Human Performance, 11*(4), 321–335.

Niemiec, R. P., Sikorski, C., & Walberg, H. J. (1996). Learner-control effects: A review of reviews and a meta-analysis. *Journal of Educational Computing Research, 15*(2), 157–174.

Nunnery, J. A., Chappell, S., & Arnold, P. (2013). A meta-analysis of a cooperative learning models effects on student achievement in mathematics. *Cypriot Journal of Educational Sciences*, *8*(1), 34-48.

O’Mara, A. J., Marsh, H. W., Craven, R. G., & Debus, R. L. (2006). Do self-concept interventions make a difference? A synergistic blend of construct validation and meta-analysis. *Educational Psychologist, 41*(3), 181–206.

Othman, N. (1996). *The effects of cooperative learning and traditional mathematics instruction in grades* *K-12: A meta-analysis of findings.* Unpublished Ed.D., West Virginia University, WV.

Parsons, J. A. (1992). *A meta-analysis of learner control in computer-based learning environments.* Unpublished doctoral dissertation, Nova University, Florida.

Patall, E. A., Cooper, H. M., & Robinson, J. C. (2008). The effects of choice on intrinsic motivation and related outcomes: A meta-analysis of research findings. *Psychological Bulletin, 134*(2), 270–300.

Petscher, Y. (2010). A meta‐analysis of the relationship between student attitudes towards reading and achievement in reading. *Journal of Research in Reading*, *33*(4), 335-355.

Phelps, R. P. (2012). The effect of testing on student achievement, 1910–2010. *International Journal of Testing*, *12*(1), 21-43.

Phillips, G.W. (1983). *Learning the conservation concept: A meta-analysis.* Unpublished doctoral dissertation, University of Kentucky, KY.

Piburn, M. D. (1993, April). *Evidence from meta-analysis for an expertise model of achievement in science.* Paper presented at the Annual Meeting of the National Association for Research in Science Teaching, Atlanta, GA.

Platz, F., Kopiez, R., Lehmann, A. C., & Wolf, A. (2014). The influence of deliberate practice on musical achievement: a meta-analysis. *Frontiers in Psychology*, *5*, 1-13.

Powers, D. E. (1986). Relations of test item characteristics to test preparation/test practice effects: a quantitative summary. *Psychological Bulletin, 100*(1), 67–77.

Preckel, F., Lipnevich, A. A., Boehme, K., Brandner, L., Georgi, K., Könen, T., Mursin, K., & Roberts, R. D. (2013). Morningness‐eveningness and educational outcomes: the lark has an advantage over the owl at high school. *British Journal of Educational Psychology*, *83*(1), 114-134.

Preiss, R. W., & Gayle, B. M. (2006). A meta-analysis of the educational benefits of employing advanced organizers. In B. M. Gayle, R.W. Preiss, N. Burrell & M. Allen (Eds.), *Classroom communication and instructional processes: Advances through meta-analysis* (pp. 329–344). Mahwah, NJ: Lawrence Erlbaum Associates.

Purdie, N., & Hattie, J. A. C. (1999).The relationship between study skills and learning outcomes: A meta-analysis. *Australian Journal of Education, 43*(1), 72–86.

Puzio, K., & Colby, G.T. (2013). Cooperative learning and literacy: A meta-analytic review. Journal of *Research on Educational Effectiveness, 6*, 339-360.

Ragosta, P. (2010). *The effectiveness of intervention programs to help college students acquire self-regulated learning strategies: A meta-analysis.* City University of New York.

Rayner, V., Bernard, R., & Osana, H.P. (2013). *A meta-analysis of transfer of learning in mathematics with a focus on teaching interventions.* Paper presented at the annual meeting of the American Educational Research Association, San Francisco, CA.

Richardson, M., Abraham, C., & Bond, R. (2012). Psychological correlates of university students' academic performance: a systematic review and meta-analysis. *Psychological Bulletin, 138(2),* 353.

Robbins, S. B., Lauver, K., Le, H., Davis, D., Langley, R., & Carlstrom, A. (2004). Do psychosocial and study skill factors predict college outcomes? A meta-analysis. *Psychological Bulletin, 130*(2), 261–288.

Rock, S. L. (1985). *A meta-analysis of self-instructional training research.* Unpublished doctoral dissertation, University of Illinois at Urbana-Champaign, IL.

Rohrbeck, C. A., Ginsburg-Block, M. D., Fantuzzo, J. W., & Miller, T. R. (2003). Peer-assisted learning interventions with elementary school studies: A meta-analytic review. *Journal of Educational Psychology, 95*(2), 240–257.

Rolheiser-Bennett, N. C. (1986). *Four models of teaching: A meta-analysis of student outcomes.* Unpublished doctoral dissertation, University of Oregon, OR.

Rolland, R. G. (2012). Synthesizing the evidence on classroom goal structures in middle and secondary schools a meta-analysis and narrative review. *Review of Educational Research*, *82*(4), 396-435.

Romero, C. C. (2009). *Cooperative learning instruction and science achievement for secondary and early post-secondary students: A systematic review*. Unpublished doctoral dissertation, Colorado State University.

Rowland, C. A. (2014). The effect of testing versus restudy on retention: A meta-analytic review of the testing effect. *Psychological Bulletin, 140 (6),* 1432-1463.

Rummel, A., & Feinberg, R. (1988). Cognitive evaluation theory: A meta-analytic review of the literature. *Social Behavior and Personality: An International Journal, 16*(2), 147–164.

Runyan, G. B. (1987). *Effects of the mnemonic-keyword method on recalling verbal information: A meta-analysis.* Unpublished doctoral dissertation, The Florida State University, Florida, United States.

Samson, G. E. (1985). Effects of training in test-taking skills on achievement test performance: A quantitative synthesis. *Journal of Educational Research, 78*(5), 261–266.

Schiefele, U., Krapp, A., & Schreyer, I. (1993). Metaanalyse des Zusammenhangs von Interesse und schulischer Leistung [Meta-analysis of the relation between interest and academic achievement]. *Zeitschrift für Entwicklungspsychologie und Pädagogische Psychologie, 25*, 120–148.

Schimmel, B. J. (1983, April). *A meta-analysis of feedback to learners in computerized and programmed instruction.* Paper presented at the Annual Meeting of the American Educational Research Association Montreal, Canada.

Schuler, H., Funke, U., & Baron-Boldt, J. (1990). Predictive validity of school grades: A meta-analysis. *Applied Psychology: An International Review, 39*(1), 89-103.

Scruggs, T. E., White, K. R., & Bennion, C. (1986). Teaching test-taking skills to elementary-grade students: A meta-analysis. *Elementary School Journal, 87*(1), 69–82.

Sedlmeier, P., Eberth, J., Schwarz, M., Zimmermann, D., Haarig, F., Jaeger, S., & Kunze, S. (2012). The psychological effects of meditation: A meta-analysis. *Psychological Bulletin, 138(6),* 1139-1145.

Seipp, B. (1991). Anxiety and academic performance: A meta-analysis of findings. *Anxiety, Stress, and* *Coping, 4*(1), 27–41.

Shulruf, B., Keuskamp, D., & Timperley, H. (2006). *Coursetaking or subject choice?* (No. Technical Report #7). Auckland, New Zealand: Starpath: Project for Tertiary Participation and Support, The University of Auckland.

Sibley, B.A., & Etnier, J. L. (2003).The relationship between physical activity and cognition in children: A meta-analysis. *Pediatric Exercise Science, 15*(3), 243–256.

Sitzmann, T., & Ely, K. (2011). A meta-analysis of self-regulated learning in work-related training and educational attainment: what we know and where we need to go. *Psychological Bulletin*, *137*(3), 421-441.

Skiba, R. J., Casey, A., & Center, B. A. (1985). Nonaversive procedures in the treatment of classroom behavior problems. *Journal of Special Education, 19*(4), 459–481.

Spuler, F. B. (1993). *A meta-analysis of the relative effectiveness of two cooperative learning models in increasing mathematics achievement.* Unpublished doctoral dissertation, Old Dominion University, VA.

Standley, J. M. (1996). A meta-analysis on the effects of music as reinforcement for education/ therapy objectives. *Journal of Research in Music Education, 44*(2), 105–133.

Stevens, R. J., & Slavin, R. E. (1990). When cooperative learning improves the achievement of students with mild disabilities: A response to Tateyama-Sniezek. *Exceptional Children, 57*(3), 276–280.

Stone, C. L. (1983). A meta-analysis of advance organizer studies. *Journal of Experimental Education,* *51*(4), 194–199.

Stoner, D. A. (2004). *The effects of cooperative learning strategies on mathematics achievement among middle-grades students: A meta-analysis*. Unpublished doctoral dissertation, University of Georgia.

Suri, H. (1997). *Comprehensive Review of Research on Cooperative Learning in Secondary Mathematics: A Pilot Study.* La Trobe University, Australia.

Swanson, H. L., & Lussier, C. M. (2001). A selective synthesis of the experimental literature on dynamic assessment. *Review of Educational Research, 71*(2), 321–363.

Swanson, H. L. (2001). Research on interventions for adolescents with learning disabilities: A meta- analysis of outcomes related to higher-order processing. *The Elementary School Journal, 101*(3), 331–348.

Taylor, G. (1995). Relationship between mathematics anxiety and achievement in mathematics: A meta-analysis. Unpublished doctoral dissertation, University of Ottawa.

Tenenbaum, G., & Goldring, E. (1989). A meta-analysis of the effect of enhanced instruction: Cues, participation, reinforcement and feedback, and correctives on motor skill learning. *Journal of Research and Development in Education, 22*(3), 53–64.

Tocanis, R., Ferguson-Hessler, M.G.M., & Broekkamp, H.(2001). Teaching science problem solving: An overview of experimental work. *Journal of Research in Science Teaching, 38 (4),* 442-468.

Tonetti, L., Natale, V., & Randler, C. (2015). Association between circadian preference and academic achievement: A systematic review and meta-analysis. *Chronobiology International, 32(6),* 792-801.

Trapmann, S., Hell, B.,Weigand, S., & Schuler, H. (2007). DieValidität von Schulnoten zurVorhersage des Studienerfolgs – eine Metaanalyse [The validity of school grades for academic achievement-a meta-analysis]. *Zeitschrift für Pädagogische Psychologie, 21*(1), 11–27.

Travlos, A. K., & Pratt, J. (1995). Temporal locus of knowledge of results: A meta-analytic review. *Perceptual and Motor Skills, 80*(1), 3–14.

Tubbs, M. E. (1986). Goal setting: A meta-analytic examination of the empirical evidence. *Journal of* *Applied Psychology, 71*(3), 474–483.

Uguroglu, M. E., & Walberg, H. J. (1979). Motivation and achievement: A quantitative synthesis. *American Educational Research Journal, 16*(4), 375–389.

Valentine, J. C., DuBois, D. L., & Cooper, H. M. (2004). The relation between self-beliefs and academic achievement: A meta-analytic review. *Educational Psychologist, 39*(2), 111–133.

Van Yperen, N. W., Blaga, M., & Postmes, T. (2015). A meta-analysis of the impact of situationally induced achievement goals on task performance. *Human Performance*, *28*(2), 1-16.

Vásquez, O. V., & Caraballo, J. N. (1993, August). *Meta-analysis of the effectiveness of concept mapping as a learning strategy in science education.* Paper presented at the Third International Seminar on the Misconceptions and Educational Strategies in Science and Mathematics Education, Ithaca, New York.

Verburgh, L., Konigs, M., Scherder, E.J.A., & Oosterlaan, J. (2014). Physical activity and executive functions in preadolescent children, adolescents and young adults: A meta-analysis. British Journal of Sports Medicine, 48, 973-979.

Wagner, E., & Szamosközi, S. (2012). Effects of direct academic motivation-enhancing intervention programs: A meta-analysis. *Journal of Evidence-Based Psychotherapies*, *12*(1), 85-94.

Walberg, H. J. (1982). What makes schooling effective. *Contemporary Education, 1*, 23-24.

Whitley, B. E., Jr., & Frieze, I. H. (1985). Children’s casual attributions for success and failure in achievement settings: A meta-analysis. *Journal of Educational Psychology, 77*(5), 608–616.

Wickline, V. B. (2003, August). *Ethnic differences in the self-esteem/academic achievement relationship: A meta-analysis.* Paper presented at the Annual Conference of the American Psychological Association, Toronto, ON, Canada.

Wiersma, U. J. (1992). The effects of extrinsic rewards in intrinsic motivation: A meta-analysis. *Journal of Occupational and Organizational Psychology, 65*(2), 101–114.

Wilkinson, S.S. (1980). *The relationship of teacher praise and student achievement: A meta-analysis of selected research.* Unpublished Ed.D., University of Florida, FL.

Williams, S.M. (2009). *The impact of collaborative, scaffolded learning in K-12 schools: A meta-analysis*. Cisco, Metiri, California, US.

Willson, V. L. (1983). A meta-analysis of the relationship between science achievement and science attitude: Kindergarten through college. *Journal of Research in Science Teaching, 20*(9), 839–850.

Witt, E. A. (1993, April). *Meta-analysis and the effects of coaching for aptitude tests.* Paper presented at the Annual Meeting of the American Educational Research Association, Atlanta, GA.

Witt, P. L., Wheeless, L. R., & Allen, M. (2006). A relationship between teacher immediacy and student learning: A meta-analysis. In B. M. Gayle, R.W. Preiss, N. Burrell & M.Allen (Eds.), *Classroom communication and instructional processes: Advances through meta-analysis* (pp. 149–168). Mahwah, NJ: Lawrence Erlbaum Associates.

Wittwer, J., & Renkl, A. (2010). How effective are instructional explanations in example-based learning? A meta-analytic review. *Educational Psychology Review*, *22*(4), 393-409.

Wood, R. E., Mento, A. J., & Locke, E. A. (1987). Task complexity as a moderator of goal effects: A meta-analysis. *Journal of Applied Psychology, 72*(3), 416–425.

Wright, P. M. (1990). Operationalization of goal difficulty as a moderator of the goal difficulty- performance relationship. *Journal of Applied Psychology, 75*(3), 227–234.

Xin, Y. P., & Jitendra, A. K. (1999).The effects of instruction in solving mathematical word problems for students with learning problems: A meta-analysis. *Journal of Special Education, 32*(4), 207.

Yeany, R. H., & Miller, P. A. (1983). Effects of diagnostic/remedial instruction on science learning: A meta-analysis. *Journal of Research in Science Teaching, 20*(1), 19–26.

Zenner, C., Hermeben-Kurz, S., & Walach, H. (2014). Mindfulness-based interventions in schools – a systematic review and meta-analysis. Frontiers in Psychology, 5, 1-20.

Zheng, X., Flynn, L., & Swanson, H.L. (2011). *Meta-analysis of experimental intervention studies on problem solving with math disabilities.* Paper presented at the annual conference of the American Educational Research Association.

Zoogman, S., Goldberg, S. B., Hoyt, W. T., & Miller, L. (2014). Mindfulness interventions with youth: A meta-analysis. *Mindfulness*, *6*(2), 290-302.
